# Smaller species but larger stages: Warming effects on inter‐ and intraspecific community size structure

**DOI:** 10.1002/ecy.3699

**Published:** 2022-05-23

**Authors:** Wojciech Uszko, Magnus Huss, Anna Gårdmark

**Affiliations:** ^1^ Department of Aquatic Resources Swedish University of Agricultural Sciences Öregrund Sweden

**Keywords:** adult, body size, competition, diet preference, global warming, juvenile, optimum, stage, temperature, zooplankton

## Abstract

Global warming can alter size distributions of animal communities, but the contribution of size shifts within versus between species to such changes remains unknown. In particular, it is unclear if expected body size shrinkage in response to warming, observed at the interspecific level, can be used to infer similar size shifts within species. In this study, we compare warming effects on interspecific (relative species abundance) versus intraspecific (relative stage abundance) size structure of competing consumers by analyzing stage‐structured bioenergetic food web models consisting of one or two consumer species and two resources, parameterized for pelagic plankton. Varying composition and temperature and body size dependencies in these models, we predicted interspecific versus intraspecific size structure across temperature. We found that warming shifted community size structure toward dominance of smaller species, in line with empirical evidence summarized in our review of 136 literature studies. However, this result emerged only given a size–temperature interaction favoring small over large individuals in warm environments. In contrast, the same mechanism caused an intraspecific shift toward dominance of larger (adult) stages, reconciling disparate observations of size responses within and across zooplankton species in the literature. As the empirical evidence for warming‐driven stage shifts is scarce and equivocal, we call for more experimental studies on intraspecific size changes with warming. Understanding the global warming impacts on animal communities requires that we consider and quantify the relative importance of mechanisms concurrently shaping size distributions within and among species.

## INTRODUCTION

The reduction in mean body size of organisms has been claimed as a universal response to global warming (Daufresne et al., 2009; Gardner et al., 2011; Ohlberger, 2013). As processes such as growth, feeding, reproduction, and mortality scale with both body size (Brose et al., 2006; Brown et al., 2004) and temperature (Dell et al., 2011; Vucic‐Pestic et al., 2010), warming‐driven shrinking can alter individual physiology as well as species interactions. Such size‐specific effects of warming can therefore have far‐reaching consequences for food web stability (Lindmark et al., 2019; Osmond et al., 2017) and ecosystem functioning (Gibert & DeLong, 2014; Petchey et al., 1999).

Warming‐driven shifts toward smaller animal size can arise via mechanisms acting on different levels of biological organization (Daufresne et al., 2009; Ohlberger, 2013). First, temperature typically increases the growth and development rates of small individuals, while decreasing maturation size, and leading to smaller size‐at‐age of (old) adults. Second, warming can alter the size structure within a population by regulating the ratio of small juveniles to large adults. Third, increased temperature can benefit smaller relative to larger species in a community, causing shifts in species composition. Reduced size at older age (the temperature‐size rule) has received considerable attention (Angilletta Jr. & Dunham, 2003; Atkinson, 1994; Walters & Hassall, 2006), with various physiological processes (e.g., oxygen limitation in the aquatic realm; Audzijonyte et al., 2019; Forster et al., 2012) suggested as potential mechanisms. Processes by which warming affects size structure at the community level have, however, not been explored to the same extent (but please refer to Blanchard et al., 2012; O'Connor et al., 2009; Yvon‐Durocher et al., 2011). In particular, we still lack a thorough understanding of how shifts in community size composition can arise through multiple mechanisms acting simultaneously at the individual, population, and community levels.

Interspecific and intraspecific resource competition is an important process for structuring consumer assemblages (Grover, 2002; Persson, 1985; Tilman, 1982), and depends on the overlap in resource use (Finkel & Snyder, 2008; Schoener, 1974). The more similar the feeding niches, the stronger the impact of competitive differences between consumers. Moreover, competition is governed by rates of resource productivity, consumer feeding, and metabolism. These, in turn, are strongly temperature and body size dependent, suggesting that warming can alter the competitive rank hierarchy, leading to changes in community size structure (Bestion et al., 2018; Winder et al., 2009). However, the consequences of warming effects depend on body size, and how they lead to size distribution shifts of competing species remains largely unknown, because the majority of studies on community responses to warming has focused on predator–prey interactions (Gibert & DeLong, 2014; Lindmark et al., 2019; Osmond et al., 2017).

According to the metabolic theory of ecology (MTE), individual rates of feeding, metabolism, and mortality scale exponentially with both body size and temperature (Brown et al., 2004). A universal exponential rate increase with temperature has been challenged, as rates governing consumption and growth have been found to be unimodal functions of temperature (Englund et al., 2011; Lindmark et al., 2022; Uiterwaal & DeLong, 2020). The MTE additionally assumes that body size and temperature influence biological rates independently. However, recent studies have suggested that temperature effects can be size specific, meaning that differences in body size exacerbate the differences in temperature scaling of vital rates (Killen et al., 2010; Lindmark et al., 2022; Ohlberger et al., 2012; Verberk & Atkinson, 2013). Examples include the increased allometric exponent of metabolism at high temperatures (Ikeda et al., 2001; Lindmark et al., 2018; Ohlberger et al., 2012), and the lower temperature optimum of feeding or growth with increasing size between (Angilletta Jr. et al., 2004) and within (Lindmark et al., 2022) species. Such size‐dependent warming effects that favor smaller, relative to larger, individuals are likely to lead to a shift toward smaller bodied species. Size‐dependent temperature effects on physiological rates could, however, lead to the opposite response within populations: a shift in dominance toward larger individuals with warming. Typically, juveniles and adults differ in their ability to compete for shared resources, and therefore in their net biomass production rates that would affect maturation and reproduction (de Roos & Persson, 2013). When adults are competitively dominant, a slow juvenile maturation rate limits population growth, and biomass builds up in the competitively inferior juvenile stage. However, if warming affects the biomass production rate of larger individuals more negatively than that of smaller individuals, the competitive rank hierarchy will change in favor of juveniles. Reproduction rate will then decrease and population biomass will instead be dominated by adults (Lindmark et al., 2018). Therefore, intraspecific size responses to warming need not follow the expectation (commonly inferred from interspecific studies) of size shrinking with increasing temperature (O'Connor et al., 2009; Yvon‐Durocher et al., 2011). This may have profound effects on food web structure (Reichstein et al., 2015; Schröder et al., 2005) and energy flows (Ohlberger et al., 2011).

How size‐dependent warming effects emerge concurrently within and among species is therefore poorly understood, and the robust predictions of how they affect the communities of competing consumers is lacking (but see Lindmark et al., 2018 and Ohlberger et al., 2011 for consumer‐resource pairs, and Lindmark et al., 2019 for food chains). In this study, we identified the mechanisms that cause warming‐driven shifts in population and community size structure (i.e., without accounting for individual‐level changes in size) using generic temperature‐dependent dynamic bioenergetic models, parameterized for small food webs with plankton grazers competing for algal food, and accounting for body size differences and dependencies within and among species. Our models predicted opposite size composition shifts within (from small to large stage) and between (from large to small) species due to warming, offering an explanation for the disparate observations of size shifts in zooplankton size structure.

## METHODS

### Overview

**FIGURE 1:**
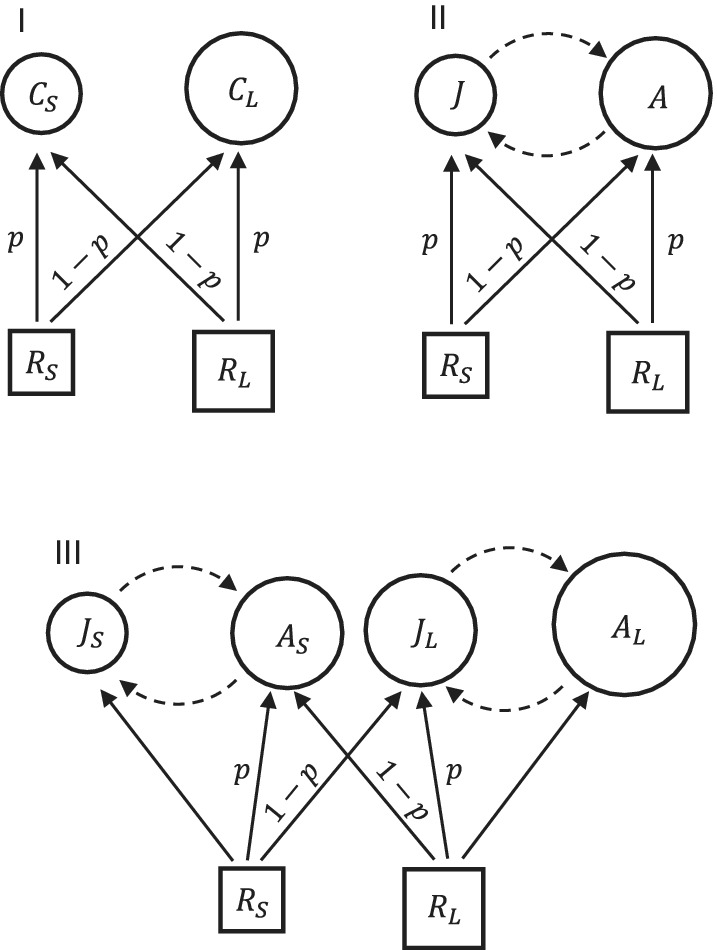
Three modeled communities. I: Two unstructured consumer species feeding on two resources. II: One stage‐structured consumer species feeding on two resources. III: Two stage‐structured consumer species feeding on two resources. Squares and circles represent different biomass compartments of food webs, and are labeled: A and $A_{i}$: adult; $C_{i}$: consumer; J and $J_{i}$: juvenile; $R_{L}$: large resource; $R_{S}$: small resource. Different sizes of circles symbolize different body masses of consumers. Solid arrows between squares and circles represent feeding links pointing in the direction of biomass flows, with the parameter p indicating the diet preference (=feeding niche dissimilarity between competing consumers; 0.5–1 in I and II, 0–1 in III). Dashed arrows between circles represent biomass flows between consumer stages related to maturation and reproduction

To identify the mechanisms causing warming‐driven shifts in population and community size structure, we carried out two sets of analyses. First, we explored how population stage structure changed model predictions compared with an unstructured model. Second, we contrasted models in which body size and temperature effects on physiological and ecological rates were independent, with models in which the strength of warming effects was modulated by individual body size (i.e., temperature effects are size dependent). We constructed three models of simple food web modules (called “Communities” henceforth) with two algal resources fed upon by one or two planktonic consumers with an intraspecific stage structure present or absent (Figure 1; Communities I–III), with various size‐dependent and temperature‐dependent parameters.

Community I consists of two zooplankton consumer species (small $C_{S}$ and large $C_{L}$) competing for two algal resources (small‐celled $R_{S}$ and large‐celled $R_{L}$). Community II consists of two competing life stages—juveniles J and adults A of body sizes identical to the two species in Community I—of a single grazer species, linked by maturation and reproduction. The strength of interspecific and intraspecific competition between consumers in Communities I and II, respectively, is governed by the diet preference parameter p, taking values between 0.5 and 1, which scales the consumer feeding rate (Figure 1; I–II). For p=0.5, both species/stages feed on both resources with equal preference. With increasing p values, each consumer feeds with increasing preference on its corresponding resource (the small species/stage on the small resource, and the large species/stage on the large resource), so that when p=1 the two consumers have fully distinct resources (no resource competition). The parameter p can therefore be understood as the inverse of (per capita) competition strength, or the measure of feeding niche dissimilarity.

In Community III (Figure 1; III), which structurally combines the previous two models, two stage‐structured consumer species (small S and large L) spanning three size classes compete for resources $R_{S}$ and $R_{L}$. Stages of identical body sizes (adults $A_{S}$ of the small consumer and juveniles $J_{L}$ of the large consumer) compete for shared resources with the diet preference parameter p varying between 0 and 1. This assumption is in line with empirical observations that consumer body size determines feeding niche overlap (Knisely & Geller, 1986), similar to what is often assumed in pelagic size spectra models (Hartvig et al., 2011). As in Communities I and II, p=1 implies no feeding niche overlap (no interspecific competition). With decreasing p values, competition between species becomes stronger, whereas competition between life stages within species becomes weaker, and at p=0 the diet preferences of $A_{S}$ and $J_{L}$ are identical (no intraspecific competition).

Communities I–III are represented by differential equation systems I–III (see Appendix S1: Table S1). Ecological characteristics of each consumer species/stage is determined solely by its body mass M. Therefore, species/stages with the same body mass are dynamically equivalent and have equal rates of feeding, metabolism, and mortality per unit biomass. We constructed two versions of the models, without and with an interaction between body size and temperature effects. In both versions, some biological rates and parameters depended on both variables as assumed by the MTE. However—and unlike in the MTE—only in the second version did the temperature effects differ, depending on species/stage body size.

The three models were empirically parameterized using literature data for pelagic plankton species. Resources represented single‐celled algal species, and consumers represented mesozooplankton grazers (see Appendix S1: Table S2 for all parameter values and references). Natural plankton communities are typically more complex than in our models, with many coexisting species spanning a wide range of body sizes (Andersen et al., 2016; Boit et al., 2012). However, as we aimed to disentangle the contribution of interspecific versus intraspecific warming effects to community size shifts, we used simple models, so that size‐dependent effects on species/stages competitive abilities were not obscured by other community‐level processes and by indirect feedback loops that are common in nature. Plankton communities are particularly well suited to address our aim, as they are strongly size structured, with body size being a master trait that regulates key biological rates (Kiørboe et al., 2018). However, the generic character of our models can give qualitative insights across other empirical systems.

### Model description

We used a stage‐structured consumer‐resource biomass model (de Roos et al., 2008) with two resource species and one or two, unstructured or stage‐structured, consumer species (Figure 1; I–III). In its unstructured version, the model simplifies to the classic Rosenzweig–MacArthur model with semichemostat resource growth (Rosenzweig & MacArthur, 1963). Full model equations, as well as parameter definitions, values, units, and references, are presented in Appendix S1: Tables S1 and S2.

In all models, the resources consisted of two algal species—small $R_{S}$ and large $R_{L}$—characterized by semichemostat dynamics with the supply rate δ, and grazed upon by consumers $C_{i}$ with feeding rate $I_{C_{i}}$:

(1) $$\frac{dR_{j}}{\mathrm{dt}}=\delta \;\left(R_{j\;\max}-R_{j}\right)-I_{C_{i}}\left(R_{j}\right)\;C_{i}$$

Equation (1) does not explicitly account for competition (e.g., for light or nutrients) between the two resource species, but instead the warming effects on resource dynamics are represented as the temperature‐dependent maximum (equilibrium) resource biomass density $R_{j\;\max}$. In the absence of consumers, $R_{j\;\max}$ declines with temperature (Bernhardt et al., 2018; Savage et al., 2004; Uszko et al., 2017). In the model including a size–temperature interaction, the two resources differed in temperature sensitivity of $R_{j\;\max}$ to implicitly capture the size‐dependent temperature effects, such that $R_{L\;\max}$ declined more steeply with warming than did $R_{S\;\max}$. This assumption stems from observations of warming‐induced shifts in the algal community structure toward smaller species in experiments (Daufresne et al., 2009; Peter & Sommer, 2013; Yvon‐Durocher et al., 2011), as well as across geographic areas (Morán et al., 2010) and seasons (Winder et al., 2009) (please refer also to recent reviews by Zohary et al. (2021) and Sommer et al. (2017)). In effect, at lower temperatures the total algal biomass was dominated by the large $R_{L}$ and at higher temperatures by the small $R_{S}$, with the switch in dominance occurring at ~20°C (i.e., in the middle of the considered temperature range) in the absence of consumers (Appendix S1: Table S2; Figure S1a). In the model version with no size–temperature interaction, the temperature dependence of $R_{L\;\max}$ was the same as that of $R_{S\;\max}$ (resources are dynamically identical).

Consumers $C_{i}$ gained their biomass by feeding on resources with feeding rate $I_{C_{i}}$ and conversion efficiency $\beta_{C_{i}}$, and lost biomass through temperature‐dependent metabolism m and constant background mortality μ:

(2) $$\frac{dC_{i}}{\mathrm{dt}}=\beta_{C_{i}}\;I_{C_{i}}\;C_{i}-m_{C_{i}}\;C_{i}-\mu_{C_{i}}\;C_{i}$$

The consumers feed with a type II functional response ($I_{C_{i}}$) on the resource $R_{j}$, modeled using the Monod function:

(3) $$I_{C_{i}}=p\;\frac{I_{i\;\max}}{H_{iR_{j}}}\;R_{j}$$

with maximum ingestion rate $I_{i\;\max}$, half‐saturation constant $H_{iR_{j}}$ and the diet preference parameter p (or 1−p; see Appendix S1: Table S2 for full equations).

The consumer populations in models II and III contained juvenile J and adult A stages that were dynamically linked through food‐dependent rates of maturation, $\max\left(\gamma_{J},0\right)$, and reproduction, $\max\left(\nu_{A},0\right)$:

(4) $$\frac{\mathrm{dJ}}{\mathrm{dt}}=\max\left(\nu_{A},0\right)\;A+\nu_{J}\;J-\max\left(\gamma_{J},0\right)\;J-\mu_{J}\;J$$

(5) $$\frac{\mathrm{dA}}{\mathrm{dt}}=\max\left(\gamma_{J},0\right)\;J+\min\left(\nu_{A},0\right)\;A-\mu_{A}\;A$$

Juveniles increase in biomass with adult reproduction (if $\nu_{A}>0$) and build up or lose biomass with the biomass production rate $\nu_{J}$ (if energy gain is less than metabolic costs, $\nu_{J}<0$, they lose biomass through starvation or starvation‐driven mortality). If the maturation term $\gamma_{J}$ is positive (Appendix S1: Table S2), biomass is transferred to the adult stage. Adults can lose biomass through starvation or starvation‐driven mortality if their biomass production rate $\nu_{A}$ is negative. All net biomass production ($\nu_{A}>0$) is used for reproduction, and appears instantaneously in form of new juvenile biomass (meaning that adults can either starve or reproduce, but do not grow). If juveniles mature faster than adults reproduce, biomass accumulates in the adult stage. If the opposite occurs, biomass accumulates in the juvenile stage. The two scenarios are referred to as reproduction and maturation limitation (de Roos et al., 2013), and can be caused by unequal resource supply and/or by different competitive abilities of the two stages. Competitive superiority of juveniles leads to reproduction limitation and adult biomass dominance, and vice versa if the adults are better competitors.

### Body mass and temperature dependence

Two rate parameters describing gains and losses of consumer biomass depend on consumer dry body mass M and ambient temperature T: maximum ingestion rate $I_{\max}$ and metabolic rate m (Appendix S1: Figure S1b,c; all other parameters are body size and temperature independent; see details in Appendix S1: Table S2). Both rates increased with body mass with an allometric exponent 0.7 (Brown et al., 2004). We assumed metabolic rate m to be an exponentially increasing function of temperature, described by the Arrhenius equation with an activation energy of −0.56 eV (Brown et al., 2004; López‐Urrutia et al., 2006) for all consumers, and a maximum ingestion rate $I_{\max}$ a unimodal function of temperature (Englund et al., 2011; Uiterwaal & DeLong, 2020). Consequently, net growth rate eventually declines with warming, leading to consumer extinction at high temperatures (Fussmann et al., 2014; Uszko et al., 2017).

Consumers are characterized by three distinct dry body mass categories—0.1, 1, and 10 μg—chosen to represent common zooplankton taxa such as rotifers and different species and stages of cladocerans and copepods (see Appendix S1: Table S2 for details and references). In the model version without size‐dependent temperature effects, we assumed (1) an optimum of maximum ingestion rate $I_{\max}$ at 20°C for all consumers (Uiterwaal & DeLong, 2020), and (2) two identical resources $R_{S}$ and $R_{L}$ (i.e., with the same temperature dependence of their maximum biomass densities $R_{\max}$). We implemented a size–temperature interaction in the models as: (1) different temperature sensitivities of $R_{\max}$ of the two resource species; and (2) declining temperature optimum of $I_{\max}$ with increasing consumer body mass (as for body growth; Angilletta Jr. et al., 2004; Lindmark et al., 2022), with temperature optima of 24, 20, and 16°C for consumers of 0.1, 1, and 10 μg, respectively. This choice of temperature optima agreed with the empirically found optima of consumer feeding rates (including zooplankton; Englund et al., 2011; Uiterwaal & DeLong, 2020; Uszko et al., 2017). We explored other alternatives for temperature optima of $I_{\max}$ by varying consumer body sizes as detailed in Appendix S2. A qualitatively identical alternative to the size‐dependent temperature optimum of the maximum feeding rate is to assume that the allometric exponent of the metabolic rate m is an increasing function of temperature (Lindmark et al., 2018, 2022). We explored this possibility in Appendix S2. In all cases with a size–temperature interaction present, increasing the temperature benefitted small relative to large consumers due to higher feeding rates or lower metabolic losses at higher temperature for smaller than for larger consumers, along with a concurrent shift toward the resource preferred by smaller consumers.

### Model analysis

**FIGURE 2:**
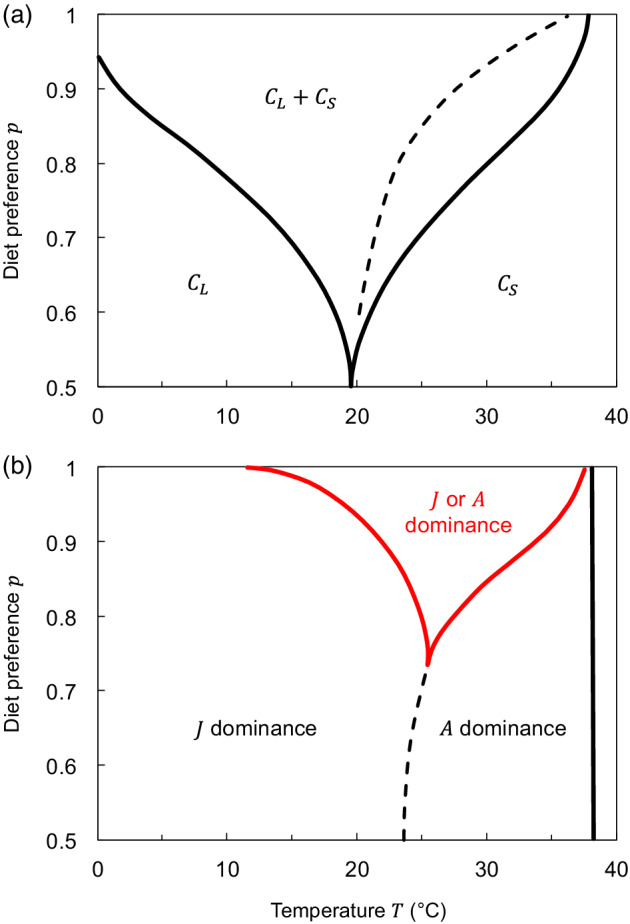
Consumer persistence (solid black lines), biomass dominance (dashed black lines), and alternative stable states (solid red lines) boundaries in temperature–diet preference space, with a size–temperature interaction present both in the maximum resource density $R_{\max}$ and in the temperature optimum of the maximum consumer ingestion rate $I_{\max}$. (a) Two unstructured consumer species feeding on two resources (Community I). (b) One stage‐structured consumer species feeding on two resources (Community II). In (a), left and right solid lines represent the persistence boundary of, respectively, consumers $C_{S}$ and $C_{L}$, that is persistence is possible above the lines (=coexistence, marked $C_{L}+C_{S}$). On the left‐hand side of the dashed line, community biomass is dominated by the large consumer $C_{L}$, and on the right‐hand side by the small consumer $C_{S}$. Consumer $C_{S}$ becomes extinct >41.2°C. In (b), the consumer extinction boundary is marked with a solid black line. All equilibria are stable

**FIGURE 3:**
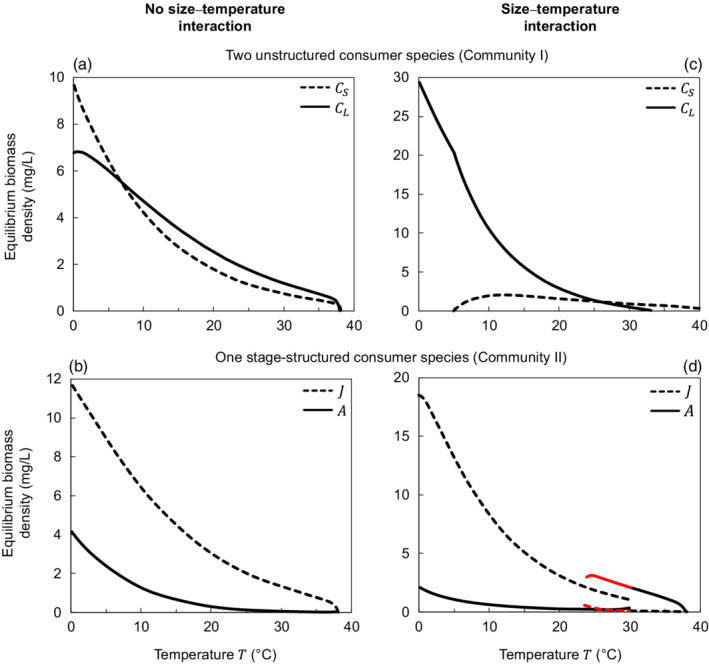
Stable‐state (equilibrium) consumer biomass densities along the temperature gradient. Left panels (a, b) show model results with no size–temperature interaction. Right panels (c, d) show model results with a size–temperature interaction present both in the maximum resource density $R_{\max}$ and in the temperature optimum of the maximum consumer ingestion rate $I_{\max}$. Upper panels (a, c) represent Community I, and lower panels (b, d) represent Community II. Red lines in panel (d) show an alternative stable state for biomass equilibrium densities. In all panels, the diet preference p=0.85

**FIGURE 4:**
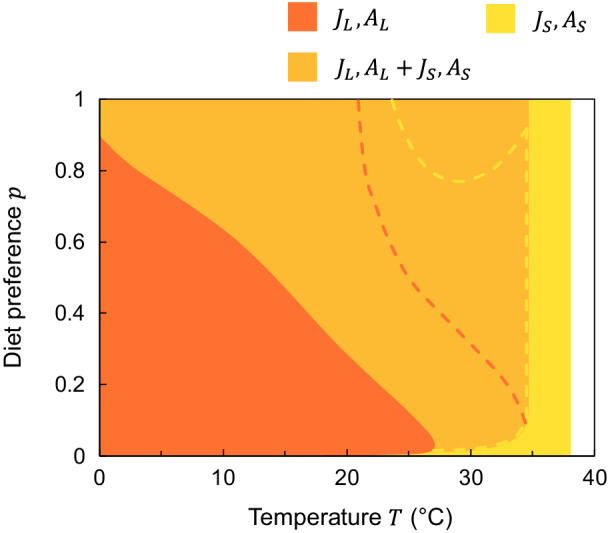
Consumer persistence regions (colored areas) and biomass dominance boundaries (dashed lines of respective colors) in temperature–diet preference space for Community III (two stage‐structured consumer species feeding on two resources). A size–temperature interaction was present both in the maximum resource density $R_{\max}$ and in the temperature optimum of the maximum consumer ingestion rate $I_{\max}$. On the left‐hand side of the dashed lines, community biomass is dominated by juveniles $J_{i}$ and on the right‐hand side by adults $A_{i}$. Note that the biomass dominance curve for the small consumer ($J_{S},A_{S}$) overlaps with the persistence boundary of the large consumer ($J_{L},A_{L}$). All equilibria are stable

We compared warming effects on consumer coexistence and size structure between models I, II, and III. Specifically, we analyzed how persistence and coexistence of consumer species and stages, including the potential for alternative stable states, depended on ambient temperature T and competition strength (represented as the diet preference parameter, p). We performed bifurcation analyses in temperature–diet preference space (T×p) to identify boundaries for persistence and alternative stable states (solid lines in Figures 2 and 3, colored regions in Figure 4). Additionally, we identified boundaries in the T×p space at which biomass dominance changes from smaller to larger species/stages or vice versa (dashed lines in Figures 2, 4). We also studied how the equilibrium biomass (Figure 3) and mean body size of consumers (Appendix S2: Figures S3 and S5) changed in the different food webs across temperature, with the diet preference p fixed for illustrative purposes at an intermediate level that yielded consumer coexistence and a relatively rich complexity of possible community states (see *Results*).

For model sensitivity analysis (Appendix S2), we explored all possible combinations of models I and II by varying the following two assumptions: (1) presence versus absence of a size–temperature interaction in maximum resource density $R_{\max}$; (2) presence versus absence of a size–temperature interaction in consumer rates, expressed either as (1) a mass‐dependent temperature optimum of the maximum ingestion rate $I_{\max}$, or as (2) a temperature‐dependent allometric exponent of the metabolic rate m (Appendix S2: Figure S4). We also altered the background mortality μ and the scaling coefficient of the temperature‐dependent maximum resource density $R_{\max}$ to test the qualitative robustness of our results (Appendix S2: Figures S1 and S2). In order to test the sensitivity of the warming effects on persistence to the relative differences in consumer body sizes, we varied the large‐to‐small consumer size ratio in models I and II (Appendix S2: Figure S6), and tested two alternative sets of body size classes in model III (Appendix S2: Figure S7). For the purpose of the sensitivity analysis, we kept the diet preference parameter p fixed at an intermediate level.

We performed bifurcation analyses using *MatCont 6p6* in MATLAB R2018b and R2021a. The model code (in MATLAB and Python) is available online at https://doi.org/10.5281/zenodo.5897520.

## RESULTS

We found different size‐structure responses to warming for between versus within species: a dominance shift toward the small species versus toward the adult stage (Figure 2). However, these effects, both within and between species in our modeled communities, occurred only when assuming an interaction between body size and temperature that favored smaller relative to larger consumers.

In Community I, with two unstructured grazers competing for two algal resources, when including size‐dependent temperature effects, warming caused a dominance shift from the large to the small consumer species (Figure 2a). When competition between the consumers was the strongest (p=0.5, complete niche overlap), coexistence was not possible, as resources were monopolized by large or small species when cold and warm, respectively. However, as the difference between the consumer diet niches was increased, the temperature region in which they coexisted increased (diverging black solid lines; Figure 2a). When coexistence was possible, warming led to a dominance shift from large to small species (dashed lines, Figure 2a). Ultimately, both species became extinct with warming, first the large $C_{L}$, then the small $C_{S}$ (Figure 2a).

The opposite occurred within a consumer species with juvenile and adult stages competing for two resources (Community II; Figure 2b) because warming caused a shift toward larger (adult) individuals. This arose from the same process as found for Community I, that is, warming benefitted small over large individuals due to a lower temperature optimum for feeding of the latter. However, as the consumer was stage structured, this caused a high maturation rate of juveniles, but low adult reproduction, leading to biomass build‐up in the adult stage. Additionally, when resource competition between stages was weak (high *p* values), a region with alternative stable states appeared (solid red lines, Figure 2b). Here either juvenile or adult biomass dominated, depending on initial abundances. However, the region with alternative states quickly disappeared with increasing background mortality μ (Appendix S2: Figure S1b).

In our models, a size–temperature interaction is a necessary condition for warming‐driven shifts toward smaller species and larger stages. In Figure 3a,b, we show the results of a “traditional” model in which the body size and temperature effects are independent (as in MTE), with moderate diet niche overlap (p=0.85). Under these assumptions, Community I is dominated by the large grazer at moderate to high temperatures (solid line, Figure 3a), and in Community II juveniles make up most of the biomass across the entire temperature range (dashed line, Figure 3b). In contrast, with size‐specific temperature effects, the smaller species and adults dominated in Community I and Community II, respectively, at high temperatures (Figure 3c,d; panels correspond to Figure 2a,b, respectively; p=0.85). Consequently, mean individual body mass decreased with warming in Community I and increased in Community II, in which temperature effects were size dependent (Appendix S2: Figure S3c,d), but stayed fairly constant in the absence of this interaction (Appendix S2: Figure S3a,b).

Warming led to a shift to smaller consumer species, but larger stages within species, also in the more complex Community III, with two stage‐structured consumers that exhibited both interspecific and intraspecific competition, with size–temperature interactions present (Figure 4). Interestingly, mean individual body mass increased with warming when resources were monopolized by a single consumer, but decreased when two consumers coexisted (Appendix S2: Figure S5a). This showed that the species shift, rather than the stage shift with warming shaped the overall mean body size of coexisting consumers in the Community III model.

## DISCUSSION

We present novel results on how warming can shape community size structure through opposite responses in body size distributions within and between species. Warming leads to a dominance shift from large to small consumer species in our models, in line with the common expectation of size shrinking in response to warming (Daufresne et al., 2009; Gardner et al., 2011; Ohlberger, 2013). However, at the intraspecific (population) level, the opposite was the case.

**FIGURE 5:**
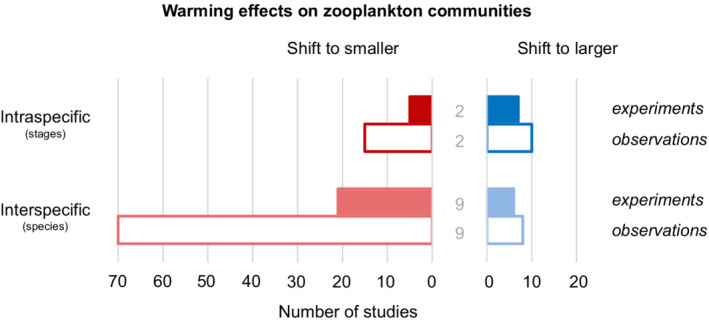
Summary of the literature review of published studies (136 articles) on warming‐induced changes in intraspecific and interspecific size structures of competitive zooplankton communities. The numbers of studies that reported the given observation (164 in total) are divided into three categories: shift to smaller species/stages (red bars), shift to larger species/stages (blue bars), or no observed effect (gray numbers between the bars). This is further divided into two levels: experimental (filled bars) and observational (empty bars). A full description of the review methods, analysis, as well as reference list and their short description, are found in Appendix S3

Natural zooplankton communities are typically characterized by more trophic links and diverse sets of consumer body sizes compared with our simple models (Boit et al., 2012). Still, our model results agreed to a large extent with the prevalent empirical evidence as summarized in a review of studies published between 1945 and 2020 that reported warming‐induced changes in size structure of competitive zooplankton communities (Figure 5). In the review, we qualitatively analyzed 164 observations from 136 articles, divided into interspecific/intraspecific and experimental/observational (see Appendix S3 for detailed methods and results, including short summaries of all included articles). Across 123 studies describing interspecific warming effects in zooplankton communities, a majority of them (91) observed a shift from larger to smaller species, and only 14 reported the opposite effect. This difference was particularly strong in the observational studies. In 18 cases there was no observable effect of warming on zooplankton size structure. By contrast, across 41 studies dealing with population stage composition, nearly half of them (20) reported a shift from larger to smaller stages with warming, and the other half found either a shift to larger stages (17 studies) or no effect of warming (four studies).

Despite the fact that many forces simultaneously influence community size structure in natural systems (Appendix S3), our relatively simple models of consumer competition, empirically parameterized for pelagic plankton, were sufficient to reproduce the expected warming effect at the species composition level. Conversely, the review results on changes in intraspecific (stage) structure caused by warming were rather equivocal (Figure 5). The insights gained from our model analyses offer an (at least partial) explanation for this lack of a clear pattern in warming‐driven stage shifts in empirical systems. That is, warming‐induced changes in stage‐specific competitive ability may lead to increasingly adult‐dominated consumer populations. As our modeling results concerned stable‐state (equilibrium) conditions, we expected them to fit particularly well with the empirical data from long‐term observations of systems characterized by relatively stable across‐season and within‐season patterns as found in, for instance, an upwelling area offshore of South Africa (Pretorius et al., 2016) and for a bay in the Barents Sea (Dvoretsky & Dvoretsky, 2009). In both cases, a dominance of adult zooplankton stages relative to juveniles coincided with warmer conditions. However, we also noted that many reviewed studies were not designed to directly address temperature effects on grazer stage structure (Appendix S3). Additionally, the empirical results may be biased due to, for instance, seasonal patterns in zooplankton reproduction (as in Kang et al., 2006; Turner, 1982), or too short experimental durations allowing for only transient changes in stage abundances (as in Beisner et al., 1997; Garzke et al., 2015). Moreover, how warming affects natural plankton communities may also depend on interactive effects and feedbacks between resource abundance, nutrient availability, and consumer physiological and ecological strategies (Diehl et al., 2022; O'Gorman et al., 2017). We argue that to understand the response of community size structure to warming, concurrent intraspecific and interspecific size structure shifts need to be accounted for. As we found a rich abundance of studies (mostly comparative across geographical areas and years; Figure 5) dealing with shifts in zooplankton species composition, we envisage a strong need for more studies, both experimental and observational, addressing warming effects on intraspecific size structure. They should involve, for instance, long‐term mesocosm, as well as laboratory, studies looking directly at stage‐specific performance across temperature (see Haubrock et al., 2020 for a recent terrestrial example).

Shrinking body size has been claimed as the “third universal response” to warming (beside shifts of species ranges and seasonal events; Gardner et al., 2011), with an intraspecific life stage shift as one of the possible mechanisms for altered community size distributions. However, our findings from both the model analyses and the literature review contrasted with the few previously reported empirical cases of an increase in the proportion of juveniles with warming (reviewed in Daufresne et al., 2009; see also Appendix S3). We find that an increase in adult abundance occurs when warming renders juveniles competitively superior, resulting in faster biomass build‐up in the older (adult) stage relative to the juvenile stage. This is likely to occur in most natural systems, as it is a direct consequence of the same mechanism that causes the ubiquitous shift toward smaller species with warming. However, other mechanisms could diminish this effect, or even cause a shift to juveniles instead, for example, temperature‐dependent stage‐specific mortality (Ohlberger et al., 2011), varying thermal tolerance ranges through ontogeny (Dahlke et al., 2020; Pörtner & Farrell, 2008), or increased energy allocation to reproduction with warming (Morgan et al., 2010; van Winkle et al., 1997). Additionally, decreased individual size with warming (as described by the temperature‐size rule; Atkinson, 1994, Ohlberger, 2013) may counteract an increase in mean individual body size driven by relatively higher abundances of larger stages. Therefore, reliable predictions of changes in size structure within species require a more thorough understanding of underlying, potentially system‐specific, physiological processes and their temperature dependencies, and how they impact ecological interactions such as competition.

A warming‐driven shift in intraspecific size structure can have major consequences for food web functioning and ecosystem services. For instance, predators or anthropogenic exploitation may specialize only in certain stages or sizes of target species. If combined with warming‐induced changes in population size structure, it may lead to some size‐specific effects that result in altered abundances and energy flow patterns through the food web, such as biomass (over)compensation or regime shifts (for examples not including warming, see Huss et al., 2014; Schröder et al., 2009; van Kooten et al., 2005). In our model, when temperature effects depend on body size, warming leads to the emergence of alternative stable states in equilibrium densities of a stage‐structured consumer. Specifically, we observed that when the two stages do not compete strongly, population biomass is predominantly locked either in the juvenile or adult stage, especially at higher temperatures, depending on the initial stage biomasses. This result is the first account known to us of warming leading to bistability due to its effects on stage‐specific competitive performance (see Lindmark et al., 2019 for other mechanisms of warming‐induced bistability). That warming can lead to alternative states is of particular importance for conservation and management. For instance, size‐selective fisheries may not only be affected by altered size distributions in targeted species due to climate warming, but also by an increased risk of sudden shifts in the abundance and structure of exploited populations (for examples not including warming, see Cury & Shannon, 2004; Mangel & Levin, 2005). Conversely, our model showed that when the background mortality rate increased, the region with alternative states quickly disappeared (Appendix S2: Figure S1b). This property of stage‐structured models has been shown when the background mortality rate is high relative to the metabolic rate (Guill, 2009). As background mortality can be a proxy of anthropogenic exploitation, the scope for alternative states in exploited systems may be limited. We also found that the region of bistability changed along a gradient of body size ratios, increasing or decreasing as the stages became more or less similar in body size, respectively (Appendix S2: Figure S6b). As ecological function and anthropogenic exploitation are highly size dependent, and therefore likely to be affected by warming‐induced shifts in size structure, it is important to recognize that such shifts may differ within versus among species.

Body size and temperature scaling of biological processes have nearly always been assumed to independently affect individual performance (Brown et al., 2004; Rall et al., 2012). However, as evident in our model analyses, such an interaction may be needed to explain observed shifts in intraspecific and interspecific size structure with warming. We implemented this interaction as a size‐dependent maximum resource density and as a size‐dependent temperature optimum of maximum consumer feeding rate. In Appendix S2, we added yet another alternative formulation for a size–temperature interaction (a temperature‐dependent allometric exponent of the metabolic rate), and we explored all combinations of these three assumptions (or lack thereof) (Appendix S2: Figure S4). Through this sensitivity analysis, a robust picture emerged: any kind of size‐specific temperature‐dependent process (or their combination) that leads to a dominance shift toward smaller species also causes a dominance shift toward larger stages (Appendix S2: Figure S4). Moreover, this result of opposite size distribution shifts within versus between species in response to warming is robust to variation in background mortality rate (as a proxy for predation or fisheries pressure; Appendix S2: Figure S1) and consumer body sizes (Appendix S2: Figures S6 and S7). Morita et al. (2010) offered another example in their growth model in which catabolic processes (energy loss through metabolism) scale steeper with body size compared with anabolic processes (energy gain from feeding), as found within fish species (Lindmark et al., 2022). The common feature of all such mechanisms is that they result in a declining temperature optimum of net consumer growth rate along body size gradients (Lindmark, 2020; Lindmark et al., 2022), which is a hallmark of a size–temperature interaction. Our model analyses and the literature review showed that ignoring such size‐dependent temperature effects impairs our understanding of warming‐driven shifts in community size structure, as the “default” assumptions of the MTE are not sufficient to explain observed size shifts with warming.

The direction of warming responses of community size distributions is governed by mechanisms that act at the levels of individuals, populations, and species assemblages simultaneously. In our most complex model community (III), the mean individual body size was primarily driven by the shift in species abundance (from larger to smaller) rather than in intraspecific structure (from juveniles to adults), suggesting a primary role of interspecific processes in shaping the size structure of entire communities with warming. The precedence of one over the other processes may, however, depend on the size differences among species relative to among stages within species. Additionally, our simplified assumptions may not be able to fully predict warming effects in natural systems, in which the species composition, individual ontogeny, and food links topology are yet more complex. Conversely, many studies looking at the warming effects on size structure of entire communities also found a decline in body size, often represented as a decreasing slope of the community size spectrum (Blanchard et al., 2009; Yvon‐Durocher et al., 2011).

In line with classic ecological theory (Tilman, 1982), we found that competitive coexistence is possible when the two model consumer species differed in their preference for resources, that is at intermediate and high values of the parameter p (Figures 2 and 4). Importantly, though, the direction of responses to warming (i.e., dominance shift from larger species and smaller stage to smaller species and larger stage) is, in all our models, independent of the consumer preference for food. However, as the diet preference itself can be temperature dependent, warming may require feeding on more energy‐ or nutrient‐rich food to balance for modified physiological processes (Boersma et al., 2016; Carreira et al., 2016; Stibor et al., 2019). This can, in turn, alter the patterns of species persistence and alleviate the negative effects of warming for some species. In our models with a temperature‐independent diet preference, this can be represented by different trajectories in the temperature–diet preference space along the temperature gradient (e.g., increasing consumer preference for its corresponding resource with warming broadens the coexistence region; Figures 2a and 4). We therefore call for more experimental studies on competitive interactions as dependent on both temperature and body size (Hart & Bychek, 2011) and how their effects emerge via intraspecific and interspecific size distributions.

## CONCLUSIONS

We show that, in the presence of size‐dependent temperature effects in competitive communities, warming can result in a shift toward smaller species but simultaneously to larger individuals within species. We also demonstrate that this is in line with empirical evidence from zooplankton communities, and show that the commonly observed between‐species size shrinking in response to warming cannot be used to infer the opposite effects observed within species. Our novel results highlight the recently recognized need to look at warming effects on size and age structure also within species to better understand and predict warming effects on entire communities (Gårdmark & Huss, 2020). We additionally show that warming can induce alternative stable states, with increased risk of abrupt shifts in population size structure if disturbed (e.g., by exploitation). It is therefore of imminent importance to identify the nature and relative significance of these mechanisms acting on individuals, populations and species assemblages to understand warming‐induced changes in community size distributions. Our findings particularly call for experiments of how temperature concurrently affects size‐specific performance within and between species. Such efforts are key for better understanding and tackling the impacts of global warming on ecological interactions and ecosystem functioning.

## AUTHOR CONTRIBUTIONS

All authors conceived and designed the study. Wojciech Uszko performed the literature review. All authors contributed to model development. Wojciech Uszko performed all model analyses, and wrote the first draft of the manuscript with substantial revisions of Anna Gårdmark and Magnus Huss. All authors gave final approval for publication.

## CONFLICT OF INTEREST

The authors declare no conflict of interest.

## Supporting information


Appendix S1

Appendix S2

Appendix S3

## Data Availability

No data were collected for this study. This article uses novel code (WUszko, 2022) available in Zenodo at https://doi.org/10.5281/zenodo.5897520.
