# Supplementary material for: Smaller species but larger stages: Warming effects on inter‐ and intraspecific community size structure
Source: Ecology. 2022 May 23;103(7):e3699. doi: 10.1002/ecy.3699 (PMC9285768; doi:10.1002/ecy.3699)
Supplement: Supplementary file 1 — Appendix S1 [file ECY-103-0-s003.pdf]

Journal name: Ecology

Manuscript title: Smaller species but larger stages: Warming effects on inter- and intraspecific community size structure

Authors: Wojciech Uszko, Magnus Huss (ORCID ID 0000-0002-5131-6000) and Anna Gårdmark (ORCID ID 0000-0003-1803-0622)

Corresponding author: Wojciech Uszko, email address: wojciech.uszko@protonmail.com

Affiliation: Department of Aquatic Resources, Swedish University of Agricultural Sciences,

Skolgatan 6, SE-742 42, Öregrund, Sweden

Appendix S1: Detailed description of model equations, its parameters, and rate formulas.

Table S1. Full set of equations for community models I, II and III. For values, definitions, units and references of the variables, rates and parameters, see Table S2 below.

Community I<br><br>Two unstructured species	Community II<br><br>One stage-structured species	Community III<br><br>Two stage-structured species
$\frac{dR_S}{dt} = \delta_{R_S}\,(R_{S\,\max} - R_S) - I_{C_S}(R_S)\,C_S - I_{C_L}(R_S)\,C_L$<br><br>$\frac{dR_L}{dt} = \delta_{R_L}\,(R_{L\,\max} - R_L) - I_{C_S}(R_L)\,C_S - I_{C_L}(R_L)\,C_L$<br><br>$\frac{dC_S}{dt} = \beta_{C_S}\,I_{C_S}\,C_S - m_{C_S}\,C_S - \mu_{C_S}\,C_S$<br><br>$\frac{dC_L}{dt} = \beta_{C_L}\,I_{C_L}\,C_L - m_{C_L}\,C_L - \mu_{C_L}\,C_L$<br><br>$I_{C_S} = \frac{p\,\frac{I_{C_S R_S\,\max}}{H_{C_S R_S}}\,R_S + (1-p)\,\frac{I_{C_S R_L\,\max}}{H_{C_S R_L}}\,R_L}{1 + \frac{p}{H_{C_S R_S}}\,R_S + \frac{(1-p)}{H_{C_S R_L}}\,R_L}$<br><br>$I_{C_L} = \frac{(1-p)\,\frac{I_{C_L R_S\,\max}}{H_{C_L R_S}}\,R_S + p\,\frac{I_{C_L R_L\,\max}}{H_{C_L R_L}}\,R_L}{1 + \frac{(1-p)}{H_{C_L R_S}}\,R_S + \frac{p}{H_{C_L R_L}}\,R_L}$	$\frac{dR_S}{dt} = \delta_{R_S}\,(R_{S\,\max} - R_S) - I_J(R_S)\,J - I_A(R_S)\,A$<br><br>$\frac{dR_L}{dt} = \delta_{R_L}\,(R_{L\,\max} - R_L) - I_J(R_L)\,J - I_A(R_L)\,A$<br><br>$\frac{dJ}{dt} = \max(\nu_A, 0)\,A + \nu_J\,J - \max(\gamma_J, 0)\,J - \mu_J\,J$<br><br>$\frac{dA}{dt} = \max(\gamma_J, 0)\,J + \min(\nu_A, 0)\,A - \mu_A\,A$<br><br>$I_J = \frac{p\,\frac{I_{J R_S\,\max}}{H_{J R_S}}\,R_S + (1-p)\,\frac{I_{J R_L\,\max}}{H_{J R_L}}\,R_L}{1 + \frac{p}{H_{J R_S}}\,R_S + \frac{(1-p)}{H_{J R_L}}\,R_L}$<br><br>$I_A = \frac{(1-p)\,\frac{I_{A R_S\,\max}}{H_{A R_S}}\,R_S + p\,\frac{I_{A R_L\,\max}}{H_{A R_L}}\,R_L}{1 + \frac{(1-p)}{H_{A R_S}}\,R_S + \frac{p}{H_{A R_L}}\,R_L}$<br><br>$\nu_J = \beta_J\,I_J - m_J$<br><br>$\gamma_J = \frac{\nu_J - \mu_J}{1 - z_{JA}^{\,1-\frac{\mu_J}{\nu_J}}}$<br><br>$\nu_A = \beta_A\,I_A - m_A$	$\frac{dR_S}{dt} = \delta_{R_S}\,(R_{S\,\max} - R_S) - I_{J_S}(R_S)\,J_S - I_{A_S}(R_S)\,A_S - I_{J_L}(R_S)\,J_L$<br><br>$\frac{dR_L}{dt} = \delta_{R_L}\,(R_{L\,\max} - R_L) - I_{A_S}(R_L)\,A_S - I_{J_L}(R_L)\,J_L - I_{A_L}(R_L)\,A_L$<br><br>$\frac{dJ_S}{dt} = \max(\nu_{A_S}, 0)\,A_S + \nu_{J_S}\,J_S - \max(\gamma_{J_S}, 0)\,J_S - \mu_{J_S}\,J_S$<br><br>$\frac{dA_S}{dt} = \max(\gamma_{J_S}, 0)\,J_S + \min(\nu_{A_S}, 0)\,A_S - \mu_{A_S}\,A_S$<br><br>$\frac{dJ_L}{dt} = \max(\nu_{A_L}, 0)\,A_L + \nu_{J_L}\,J_L - \max(\gamma_{J_L}, 0)\,J_L - \mu_{J_L}\,J_L$<br><br>$\frac{dA_L}{dt} = \max(\gamma_{J_L}, 0)\,J_L + \min(\nu_{A_L}, 0)\,A_L - \mu_{A_L}\,A_L$<br><br>$\nu_{J_S} = \beta_{J_S}\,I_{J_S} - m_{J_S}$<br><br>$\gamma_{J_S} = \frac{\nu_{J_S} - \mu_{J_S}}{1 - z_{J_S A_S}^{\,1-\frac{\mu_{J_S}}{\nu_{J_S}}}}$<br><br>$\nu_{A_S} = \beta_{A_S}\,I_{A_S} - m_{A_S}$<br><br>$\nu_{J_L} = \beta_{J_L}\,I_{J_L} - m_{J_L}$<br><br>$\gamma_{J_L} = \frac{\nu_{J_L} - \mu_{J_L}}{1 - z_{J_L A_L}^{\,1-\frac{\mu_{J_L}}{\nu_{J_L}}}}$<br><br>$\nu_{A_L} = \beta_{A_L}\,I_{A_L} - m_{A_L}$

Table S2. The list of values, definitions, units and references of the variables, rates and parameters used in models I, II and III. The reference list is found below.

Variable or rate	Formula	Definition and unit	References
R_j	-	Resource biomass density (mg dry mass/L)	-
C_i	-	Consumer biomass density (mg dry mass/L)	-
J, J_i	-	Juvenile consumer biomass density (mg dry mass/L)	-
A, A_i	-	Adult consumer biomass density (mg dry mass/L)	-
$R_{S\,max}$	$4.2 \cdot 10^{-3} \cdot \exp\big(0.15/(k \cdot T)\big)$	Supply (maximum) R_S biomass density (mg dry mass/L)	1, 8, 12, 16
$R_{L\,max}$	$5.9 \cdot 10^{-7} \cdot \exp\big(0.38/(k \cdot T)\big)$, or $=R_{S\,max}$	Supply (maximum) R_L biomass density (mg dry mass /L)	1, 12, 16
$I_{i\,max}$	$\frac{1}{M_j} \cdot 19 \cdot {M_j}^{0.7} \cdot \exp\left(\frac{-(T-T_{opt\,i})^2}{2 \cdot s^2}\right)$	Mass-specific maximum ingestion rate of C_i (mg/[mg·day]) †	15, 16
m_i	$\frac{1}{M_i} \cdot 8.5 \cdot 10^8 \cdot {M_i}^{0.7} \cdot \exp\big(-0.56/(k \cdot T)\big)$	Mass-specific metabolic rate of C_i (1/day)	3, 8, 16

Parameter	Value	Definition and unit	
M_i	C_S, J, J_S=0.1; C_L, A, A_S, J_2=1; A_L=10	Body mass of C_i (ng dry mass in the model, here given in µg) ‡	2, 6, 9, 10, 11, 19
T	Control parameter; 273.15–313.15	Ambient temperature (K; 0–40 °C)	-
$T_{opt\,i}$	C_S, J, J_S=24; C_L, A, A_S, J_L=20; A_L=16, or =20 for all	Temperature optimum of $I_{i\,max}$ of C_i (K in the model, here given in °C)	13, 16, 17
s	8	Curve breadth of the temperature dependence of $I_{i\,max}$ (K)	16
k	$8.6 \cdot 10^{-5}$	Boltzmann constant (eV/K)	-

δ_{R_j}	0.1	R_j supply (inflow) rate (1/day)	4, 5, 7
β_i	0.6	C_i conversion efficiency (unitless)	14, 15
p	Control parameter; 0–1	Diet preference (unitless)	-
H_{iR_j}	0.2	Half-saturation constant of C_i on R_j (mg dry mass/L) †	15, 16
z_{kl}	0.1	Juvenile k to adult l body mass ratio (unitless)	2, 6, 9, 10, 11, 19
μ_i	0.01	Background mortality of C_i (1/day) ♠	5, 18

Footnotes

† We used the Monod instead of the Holling function, as the former allows to assume temperature dependence of only a single parameter – in our case the maximum ingestion rate I_{max} – while keeping the half-saturation constant H body size- and temperature-independent (Fussmann et al. 2014, Mulder and Hendriks 2014). As the half-saturation constant is strongly correlated with the maximum ingestion rate, changing either of these two parameters affects the consumer functional response in a similar way (Kiørboe and Andersen 2019, Barraquand and Gimenez 2021).

‡ The lowest size category $M = 0.1$ µg was assumed for consumer species C_S, and consumer juveniles J and J_S in models I, II and III, respectively. This size corresponds to small planktonic grazers such as rotifers, juveniles of small crustacean species such as *Bosmina*, or naupliar stages of copepods. The medium size category $M = 1$ µg was assumed for consumer species C_L, consumer adults A, and adults A_S and juveniles J_L in models I, II and III, respectively. This corresponds to planktonic grazers such as adult *Bosmina* and juvenile (copepodite) copepod stages. The largest size category $M = 10$ µg was assumed for consumer adults A_L in model III, and corresponds to adult stages of copepods and larger crustaceans.

♠ Our formulation of the stage-structured model requires the background mortality $\mu > 0$ (see the formula for the maturation rate γ). The background mortality can represent additional sources of consumer biomass loss (apart from metabolic rate) such as predation by higher trophic levels or anthropogenic exploitation.

Here, we assume a low background mortality rate identical for all species and stages, and explore model sensitivity to changes in this parameter in Appendix S2: Fig. S1.

References

1. Bernhardt, J. R., J. M. Sunday, and M. I. O'Connor. 2018. Metabolic theory and the temperature-size rule explain the temperature dependence of population carrying capacity. American Naturalist 192:687–697.

2. Bottrell, H. H., A. Duncan, Z. M. Gliwicz, E. Grygierek, A. Herzig, A. Hillbricht-Ilkowska, H. Kurasawa, P. Larsson, and T. Węgleńska. 1976. A review of some problems in zooplankton production studies. Norwegian Journal of Zoology 24:419–456.

3. Brown, J. H., J. F. Gillooly, A. P. Allen, V. M. Savage, and G. B. West. 2004. Toward a metabolic theory of ecology. Ecology 85:1771–1789.

4. de Roos, A. M., and L Persson. 2001. Physiologically structured models - from versatile technique to ecological theory. Oikos 94:51–71.

5. de Roos, A. M., and L. Persson. 2013. Population and community ecology of ontogenetic development. Princeton University Press.

6. Dumont, H. J., I. Van de Velde, and S. Dumont. 1975. The dry weight estimate of biomass in a selection of Cladocera, Copepoda and Rotifera from the plankton, periphyton and benthos of continental waters. Oeologia 19:75–97.

7. Lindmark, M., J. Ohlberger, M. Huss, and A. Gårdmark. 2019. Size-based ecological interactions drive food web responses to climate warming. Ecology Letters 22:778–786.

8. López-Urrutia, A., E. San Martin, R. P. Harris, and X. Irigoien. 2006. Scaling the metabolic balance of the oceans. Proceedings of the National Academy of Sciences of the United States of America 103:8739–8744.

9. Miller, C. B., J. K. Johnson, and D. R. Heinle. 1977. Growth rules in the marine copepod genus *Acartia*. Limnology and Oceanography 22:326–335.

10. Pauli, H.-R. 1989. A new method to estimate individual dry weights of rotifers. Hydrobiologia 186/187:355–361.

11. Porter, K. G., Y. S. Feig, and E. F. Vetter. 1983. Morphology, flow regimes, and filtering rates of *Daphnia*, *Ceriodaphnia* and *Bosmina* fed natural bacteria. Oecologia 58:156–163.

12. Raven, J. A., R. J. Geider. 1988. Temperature and algal growth. New Phytologist 110:441–461.

13. Uiterwaal, S. F., and J. P. DeLong. 2020. Functional responses are maximized at intermediate temperatures. Ecology 101:e02975.

14. Urabe, J., and Y. Watanabe. 1991. Effect of food concentration on the assimilation and production efficiencies of *Daphnia galeata* G.O. Sars (Crustacea: Cladocera). Functional Ecology 5:635–641.

15. Uszko, W., S. Diehl, N. Pitsch, K. Lengfellner, and T. Müller. 2015. When is a type III functional response stabilizing? Theory and practice of predicting plankton dynamics under enrichment. Ecology 96:3243–3256.

16. Uszko, W., S. Diehl, G. Englund, and P. Amarasekare. 2017. Effects of warming on predator-prey interactions – a resource-based approach and a theoretical synthesis. Ecology Letters 20:513–523.

17. Uszko, W., S. Diehl, and J. Wickman. 2020. Fitting functional response surfaces to data: a best practice guide. Ecosphere, 11:e03051.

18. Wollrab, S., A. M. de Roos, and S. Diehl. 2013. Ontogenetic diet shifts promote predator-mediated coexistence. Ecology 94:2886–2897.

19. Own measurements of the body size of zooplankton species and stages from Lugol-preserved contemporary coastal samples originating from the Baltic Sea, near Forsmark 60.42 N 18.16 E, Sweden.

Barraquand, F. and O. Gimenez. 2021. Fitting stochastic predator–prey models using both population density and kill rate data. Theoretical Population Biology 138:1–27.

Fussmann, K. E., F. Schwarzmüller, U. Brose, A. Jousset, and B. C. Rall. 2014. Ecological stability in response to warming. Nature Climate Change 4:206–210.

Kiørboe, T., and K. H. Andersen. 2019. Nutrient affinity, half-saturation constants and the cost of toxin production in dinoflagellates. Ecology Letters 22:558–560.

Mulder, C., and A. J. Hendriks. 2014. Half-saturation constants in functional responses. Global Ecology and Conservation 2:161–169.

Figure S1. Temperature-dependent and body mass M-dependent rate parameters across the temperature gradient T. For parameter definitions and references, see Appendix S1: Table S2.
A: Supply (maximum) resource density (mg dry mass/L) with a size–temperature interaction present. In absence of a size-temperature interaction, for both resources their supply densities are equal to $R_{S\,max}$ (dashed line).
B1: Mass-specific maximum ingestion rate of consumers (mg resource dry mass per day per mg consumer dry mass) with a size–temperature interaction present. B2: Mass-specific maximum ingestion rate of consumers (mg resource dry mass per day per mg consumer dry mass) with a size–temperature interaction absent, i.e. the temperature optimum of I_{max} occurs at 20 °C for all consumers.
C1: Mass-specific metabolic rate of consumers (per day per mg dry mass) with a size–temperature interaction absent. C2: Mass-specific metabolic rate of consumers (per day per mg dry mass) with a size–temperature interaction present in the allometric exponent of the metabolic rate $m = \frac{1}{M} \cdot 8.5 \cdot 10^{8} \cdot M^{(0.7+0.0005 \cdot T)} \cdot \exp\left(-0.56/(k \cdot T)\right)$, where M is the consumer dry body mass (ng), and k is the Boltzmann constant. Curves from C1 (without a size-temperature interaction) are shown as thinner dashed lines. Note that each consumer suffers from higher metabolic rate in the presence of a size–temperature interaction proportionally to its size, with the largest consumer having the highest relative increase in m. For further information and results including this assumption, see Appendix S2, and Figure S4 therein.

Figure S1.

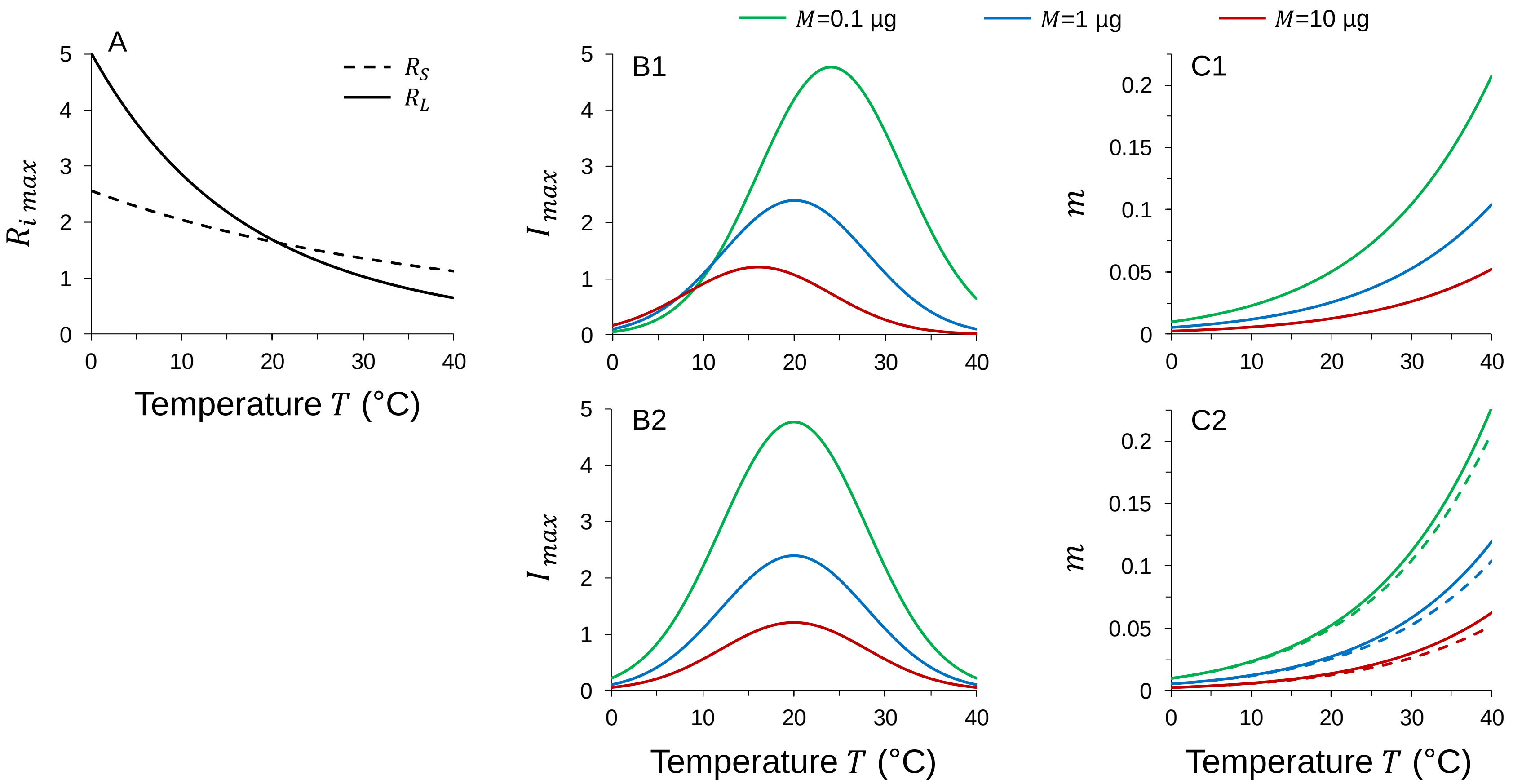

M=0.1 µg
M=1 µg
M=10 µg
A
R_S
R_L
$R_{i\,max}$
Temperature T (°C)
B1
B2
I_{max}
C1
C2
m
Temperature T (°C)
Temperature T (°C)
