# Supplementary material for: Smaller species but larger stages: Warming effects on inter‐ and intraspecific community size structure
Source: Ecology. 2022 May 23;103(7):e3699. doi: 10.1002/ecy.3699 (PMC9285768; doi:10.1002/ecy.3699)
Supplement: Supplementary file 2 — Appendix S2 [file ECY-103-0-s002.pdf]

Journal name: Ecology

Manuscript title: Smaller species but larger stages: Warming effects on inter- and intraspecific community size structure

Authors: Wojciech Uszko, Magnus Huss (ORCID ID 0000-0002-5131-6000) and Anna Gårdmark (ORCID ID 0000-0003-1803-0622)

Corresponding author: Wojciech Uszko, email address: wojciech.uszko@protonmail.com

Affiliation: Department of Aquatic Resources, Swedish University of Agricultural Sciences, Skolgatan 6, SE-742 42, Öregrund, Sweden

Appendix S2: Further results and model sensitivity analysis.

Effects of varying background mortality rate on the patterns of species' and stages' persistence and dominance across temperature

In this part of the model sensitivity analysis, we explored the effects of increasing background mortality rate μ on the temperature effects on species' and stages' persistence patterns and on the presence and extent of alternative stable states at different temperatures. Changes in external mortality rate have been shown to cause a number of phenomena in stage-structured models and natural systems, such as biomass overcompensation (De Roos et al. 2007, Nilsson et al. 2010) and emergence of bistability (Van Kooten et al. 2005, Guill 2009).

We run a bifurcation analysis, and drew persistence, biomass dominance and alternative stable states boundaries in the temperature-background mortality rate space $T \times \mu$, for community model I (two unstructured consumer species feeding on two resources) and II (one stage-structured consumer species feeding on two resources). Here, we assumed a size–temperature

interaction in both the maximum resource density R_{max} and in the temperature optimum of the maximum consumer ingestion rate I_{max}, and that the diet preference parameter $p = 0.85$. We varied the background mortality rate μ, assumed equal for all species and stages, between 0.01 and 0.5 per day. That is, the result for $\mu = 0.01$ is equivalent to the results shown in Fig. 2 (main text) with $p = 0.85$.

In community model I (two unstructured consumer species feeding on two resources, Fig. S1A), increasing the background mortality rate does not change the qualitative patterns of how temperature affects species' persistence. With increasing temperature, and regardless of the level of background mortality rate, the community goes through the following changes in structure (in order from low to high temperatures): both consumers extinct – only the large consumer C_L present – coexistence with biomass dominated by the large consumer – coexistence with biomass dominated by the small consumer C_S – only the small consumer present – both consumers extinct. However, increasing background mortality rate causes both the coexistence and the large-consumer-only regions to shrink while widening the region with only the small consumer present (solid lines, Fig. S1A). Additionally, within the coexistence region, increasing background mortality rate widens the region dominated by the small consumer (right-hand-side of the dashed line, Fig. S1A).

In community model II (one stage-structured consumer species feeding on two resources, Fig. S1B), the most striking effect of increasing background mortality rate is a quick disappearance of the alternative stable state region at $\mu > 0.03$ (solid red line, Fig. S1B). However, the general pattern persists, i.e. the consumer biomass shifts from juvenile to adult dominance with warming (dashed line, Fig. S1B). As in model I, an increase in the background mortality rate causes the consumer persistence region to shrink (solid black line, Fig. S1B).

Additionally, we have looked at the effects of altering the background mortality rate only for the large species and stage (μ_{C_L} and μ_A, respectively), with the background mortality for the small species and stage (μ_{C_S} and μ_J, respectively) kept constant at the original value of 0.01. This can be interpreted as elevated predation pressure on the more vulnerable (=larger) consumer. Also in this case, we have found that the qualitative community shifts along the temperature gradient are independent of the background mortality rate on the large species/stage. Specifically, in Community I and for the background mortality rate $\mu_{C_L} < 0.1$, warming causes a shift from presence/dominance of the large species to presence/dominance of the small species. At the background mortality rate $\mu_{C_L} > 0.1$, only the small species C_S can exist. In Community II, at all values of μ_A between 0.01 and 0.5, the consumer stage structure follows a nearly identical pattern as shown in Fig. S1 B – the alternative stable state region quickly disappears with increasing μ_A, and the community shifts from juvenile to adult dominance with warming.

We have also investigated the effects of increasing background mortality rate only for the small species and stage (μ_{C_S} and μ_J, respectively), with the background mortality for the large species and stage (μ_{C_L} and μ_A, respectively) kept constant at the original value of 0.01. This scenario, contrary to the one above, can be interpreted as elevated predation pressure on the small consumer. This could potentially counteract the patterns found in the original model as now the small consumer – benefitted by warming – experiences higher mortality losses. However, also here we have found that the qualitative pattern of community shifts along the temperature gradient are independent of the background mortality rate on the small species/stage. Specifically, Community I shifts with warming, at all values of the background mortality rate μ_{C_S}, from presence of only the large species C_L to coexistence of both species (with dominance shift from C_L to the small species C_S) to extinction of C_L followed by extinction of C_S. The large species C_L,

however, does not go extinct at low temperatures at high values of μ_{C_S}, and the coexistence region is shifted towards higher temperatures compared to the original model version. In Community II, the qualitative pattern remains the same as in the original model for all values of the background mortality rate μ_J – the community shifts with warming from extinction to persistence with juveniles dominating, followed by dominance shift to adults and species extinction, and the alternative stable state region disappears for $\mu_J > 0.1$. Interestingly, the juvenile-to-adult dominance shift happens at increasingly lower temperature with increasing μ_J, and for $\mu_J > 0.45$ the community is dominated by adults at all temperatures.

Effects of varying resource enrichment on the patterns of species' and stages' persistence and dominance across temperature

In this part of the model sensitivity analysis, we explored the effects of resource enrichment on how temperature affects patterns of species' and stages' persistence and the presence of a region with alternative stable states. The resource enrichment level is generally known to strongly influence the patterns of species' persistence and stability across temperature, leading to various dynamic consequences of warming on modeled food webs (Uszko et al. 2017). Here we represent and modify the resource enrichment as the coefficient R_0, which scales the default formula of temperature dependence of the maximum (supply) resource biomass densities, i.e., $R_0 \cdot R_{max}(T)$ (see Appendix S1: Table S2).

We run a bifurcation analysis, and drew persistence, biomass dominance and alternative stable states boundaries in the temperature-scaling coefficient space $T \times R_0$, for community model I (two unstructured consumer species feeding on two resources) and II (one stage-structured consumer species feeding on two resources). Here, we assumed a size–temperature

interaction in both the maximum resource density R_{max} and in the temperature optimum of the maximum consumer ingestion rate I_{max}, and the diet preference parameter $p = 0.85$. We varied the scaling coefficient R_0, assumed equal for both resources (R_S and R_L), between 0 and 3. That is, the result for $R_0 = 1$ is equivalent to the results shown in Fig. 2 (main text) with $p = 0.85$.

We find that the patterns of species and stages persistence, dominance and alternative stable states over temperature are very robust to changes in resource enrichment, both in community models I (Fig. S2A) and II (Fig. S2B). In other words, the temperatures representing species persistence boundaries (solid black lines, Fig. S2) and alternative stable states boundaries (solid red lines, Fig. S2B) are nearly insensitive to changes in the scaling coefficient R_0 across most of the considered values. Other than that, the general pattern of species persistence across temperature follows the expected U-shaped pattern (Uszko et al. 2017). That is, consumer species go extinct at both very low and very high temperatures because of too low feeding rates and too high metabolic rates, respectively. Similarly, consumers cannot persist at extremely low resource enrichment values. Interestingly, we found no shift from stable equilibria to limit cycles (Hopf bifurcation) across the considered range of temperature and scaling coefficient R_0. Even though oscillations should, in principle always arise at sufficiently high enrichment when consumers feed with a type II functional response, the actual dynamic behavior depends on several parameters of consumer and resource growth, feeding and mortality (Uszko et al. 2015).

Effects of warming on mean individual consumer body size

Here, we present the results concerning warming effects on mean individual body size in models I (two unstructured consumer species feeding on two resources, Fig. S3A, C) and II (one stage-structured consumer species feeding on two resources, Fig. S3B, D) with (Fig. S3C, D) and

without (Fig. S3A, B) a size–temperature interaction in the maximum resource density R_{max} and in the temperature optimum of the maximum consumer ingestion rate I_{max}. We do it also for model III (two stage-structured consumer species feeding on two resources (Fig. S5) with a size–temperature interaction present both in the maximum resource density R_{max} and in the temperature optimum of the maximum consumer ingestion rate I_{max}. For models I and II, the diet preference parameter is set to $p = 0.85$, and for model III $p = 0.75$. Even though we assumed fixed body masses of all species and stages in all our models, the mean individual body mass in the community can vary due to changes in relative proportions of species and stages of different body masses.

This exercise clearly shows that mean individual body size changes considerably only when a size–temperature interaction is present. In absence of such an interaction, mean consumer body size stays relatively constant across the entire temperature range studied in both models I and II (Fig. S3A and B, respectively). Only when a size–temperature interaction is present, mean individual body size in model I either stays constant (when the community is dominated by a single consumer species) or declines (when the two consumers coexist) with warming (Fig. S3C). The latter is caused by the shift in biomass dominance from the large to the small consumer species (compare with Fig. 2A in the main text when $p = 0.85$). In model II, the mean individual body size shifts from smaller to larger due to a shift in biomass dominance from juveniles to adults (Fig. S3D, compare with Fig. 2B in the main text when $p = 0.85$). In the temperature range of approximately 24–30 °C, the mean size depends on the initial conditions (alternative stable states, solid red lines in Fig. S3D, compare with Fig. 2B in the main text when $p = 0.85$).

In model III, mean individual body size follows a qualitatively similar pattern (Fig. S5A). When only a single stage-structured consumer is present in the community, the mean size

increases with warming due to a shift in intraspecific stage structure towards adult dominance (red and yellow regions, Fig. S3). When the two stage-structured consumers coexist, the shift of dominance towards the smaller consumer species drives the decrease in the mean individual body size, regardless of the simultaneous shift in intraspecific structure in the opposite direction (Fig. S5A, compare with Fig. 4 in the main text when $p = 0.75$).

Effects of absence vs. presence and nature of a size–temperature interaction on temperature effects on species' and stages' persistence and dominance

Here, we looked into all 12 cases of different combinations of the assumptions of absence vs. presence of a size–temperature interaction, and of the nature of this interaction. We did this for two main reasons. First, we wanted to see if the 'traditional' assumption of a lack of such an interaction (i.e., size and temperature effects are independent, as in the metabolic theory of ecology (Brown et al. 2004)) can reproduce the empirically observed patterns of shifts in species dominance with warming (see Appendix S3). Second, as part of the sensitivity analysis, we wanted to check if the presence of a size–temperature interaction gives similar results depending on how this interaction is implemented in the model. To do this, we run a bifurcation analysis, and drew persistence, biomass dominance and alternative stable states boundaries in the temperature-diet preference space $T \times p$, for community model I (two unstructured consumer species feeding on two resources) and II (one stage-structured consumer species feeding on two resources).

We implemented the size–temperature interaction in the resources as different temperature sensitivities of resource supply (maximum) density R_{max}. Following the empirically observed pattern, we assumed that R_{max} is a declining function of temperature (Savage et al. 2004, Uszko

et al. 2017, Bernhardt et al. 2018), and that it declines faster for the large than for the small resource. In effect, in absence of consumers, the resource biomass is dominated by the large resource R_L at lower temperatures, and by the small resource R_S at higher temperatures, with the shift occurring around 20 °C (Winder et al. 2009, Daufresne et al. 2009, Yvon-Durocher et al. 2011). In absence of a size–temperature interaction, both resources are dynamically equivalent, and their R_{max} follows the formula for the small resource R_S. For exact parameter formulations, see Appendix S1: Table S2 and Figure S1A.

We considered two scenarios of the presence of a size–temperature interaction in the consumers. In the first one, as in the main text, we assumed that the temperature optimum of the consumer maximum ingestion rate I_{max} declines with increasing dry body mass M (see Appendix S1: Table S2 and Fig. S1B1). This follows from the observed empirical patterns of declining consumer performance with warming (Angiletta et al. 2004, Lindmark 2020). In the absence of a size–temperature interaction, all species and stages have the same temperature optimum of the maximum ingestion rate at 20 °C (Appendix S1: Fig. S1B2), and only differ in ingestion rates due to their different body masses (that influence I_{max} independently of temperature). In the second scenario, we assumed that the allometric exponent of the metabolic rate m increases with warming following the formula $0.7 + 0.0005 \cdot T$ (Appendix S1: Fig. S1C2), similar to what has been shown in some empirical systems (Ikeda et al. 2001, Lindmark et al. 2018). Consequently, larger consumers suffer from relatively higher metabolic losses at elevated temperatures. In the absence of a size–temperature interaction, all species and stages have an allometric exponent of 0.7, and only differ due to their different body masses (that influence m independently of temperature, see Appendix S1: Table S2 and Fig. S1C1). In both scenarios, warming causes lower net biomass production (growth rates) of larger relative to smaller species and stages.

Full results of these complementary analyses are shown in the 12 panels of Fig. S4. We note three major observations. First, with a 'traditional' assumption of body size and temperature independently affecting resource and consumer dynamics, the models fail to reproduce the empirically observed patterns of warming-induced shifts in consumer community size structure. In fact, in model I (Fig. S4A), warming drives the community in the opposite direction, such that persistence and dominance of the large consumer C_L becomes more likely at elevated temperature, especially when the two consumers do not compete strongly for resources (i.e., at higher diet preference p values). In model II (Fig. S4C), the biomass is completely dominated by juveniles across the entire temperature–diet preference space.

Second, the presence of a size–temperature interaction of any type breaks the above pattern. That is, an addition of this interaction in resources or in consumers, or in both, changes the model predictions – it becomes more likely that the small consumer will dominate in community I, and that the adults will dominate in community II (Fig. S4, all panels apart from A and C). Additionally, with presence of any type of a size–temperature interaction, an alternative stable state appears in the temperature–diet preference space in community II (solid red lines, Fig. S4). In this region, placed at moderate to high values of the diet preference parameter p, the dominant stage is determined by initial abundances of juveniles and adults.

Third, the presence of a size–temperature interaction in the temperature optimum of the maximum ingestion rate I_{max} vs. in the allometric scaling exponent of the metabolic rate m leads to similar outcomes, especially in community II where the patterns of alternative stable states and stage dominance are very similar (Fig. S4E-H vs. I-L). However, the two formulations differ in the exact patterns of species persistence and dominance. In case of a size–temperature interaction in the temperature optimum of I_{max}, we observed a shift towards smaller species regardless of

the competitive strength (diet preference p), with weaker competition leading to a wider coexistence region. In the case of a size–temperature interaction in the allometric exponent of m, coexistence is only possible at moderate to high diet preference p values ($p > 0.6$), that is when the two consumer species do not compete strongly for resources.

Effects of varying body size ratios of competing consumers on the patterns of species' and stages' persistence and dominance across temperature

Here we explore how varying consumer body sizes (M; see Appendix S1: Table S2) shape temperature effects on species' and stages' persistence and the presence of alternative stable states. Natural plankton systems typically consist of coexisting consumers spanning a wide body size range (Andersen et al. 2016). As body size controls metabolic and feeding rates (and the scope of their temperature dependence) in our models, altered relative size ratios of competing species/stages can potentially modify the patterns we found. In the original formulation of our model, we consider three different fixed consumer size classes (0.1 and 1 μg dry mass in models I and II, and 0.1, 1 and 10 μg dry mass in model III). Here, we relax this assumption to check the robustness of our results to different body sizes (and body size ratios) of competing consumers. Specifically, in models I and II we kept the body size of the small consumer (C_S and J, respectively) constant at 0.1 μg, and varied the body size of the large consumer (C_L and A, respectively), rendering body size ratios M_{C_L}: M_{C_S} and M_A: M_J range from 1 to 100. Note that a decrease in adult-to-juvenile body size ratio in model II may represent a decrease in size at maturation, as predicted by the temperature-size rule. In model III, we considered two alternative assumptions of consumer body sizes: (A) 1, 5 and 10, and (B) 0.001, 0.1 and 10 μg dry mass for

the three size categories of J_S, A_S and J_L, and A_L, respectively (see Fig. 1 and Appendix S1: Table S2).

We ran a bifurcation analysis, and drew persistence, biomass dominance and alternative stable states boundaries in the body size ratio-temperature space for Community I (two unstructured consumer species feeding on two resources; Fig. S6A) and II (one stage-structured consumer species feeding on two resources; Fig. S6B), with the diet preference parameter $p = 0.85$. For Community III, we did the same in the temperature-diet preference space $T \times p$ (Fig. S7). In all models, we assumed a size–temperature interaction in both the maximum resource density R_{max} and in the temperature optimum of the maximum consumer ingestion rate I_{max}. As we varied consumer body sizes, we thus also varied the temperature optimum T_{opt} (K) of maximum consumer ingestion rate I_{max} as $T_{opt} = 305.38 \cdot M^{-0.006}$, where M is the consumer dry body mass (ng). This formula yields the temperature optima of 24, 20 and 16 °C for the three size classes of 0.1, 1 and 10 µg dry body mass as assumed in the original model. Additionally, this formula describes a linearly decreasing temperature optimum with increasing consumer body size on a log-scale, as has been shown for temperature optimum of feeding or growth between (Angilletta et al. 2004) and within (Lindmark et al. 2021) species.

We find that the patterns of species and stage persistence, dominance and alternative stable states are relatively robust to changes in consumer size ratios in Communities I (Fig. S6A), II (Fig. S6B) and III (S2.7 A and B). In all three cases, we observe similar pattern to the one found in the original formulation of the models, that is, increasing temperature causes shifts of persistence and dominance from large to small species, and from small to large stages. In Community I (Fig. S6A), with increasing body size ratio, the coexistence region (between black solid lines, Fig. S6A) shrinks. This is mostly due to the large consumer C_L going extinct at lower

temperatures the larger its body size gets. Interestingly, the temperature at which biomass dominance switches from the large to the small consumer (dashed black line, Fig. S6A) stays fairly constant ~25–28 °C at all considered body size ratios. In Community II, the region of temperatures with alternative stable states (i.e. juveniles or adults dominate depending on initial abundances) shrinks with increasing body size ratio (between red solid lines, Fig. S6B). Also in this case, the consumer goes extinct at lower temperatures the larger the adults get (solid black line, Fig. S6B). However, a decreasing adult body size (i.e., size at maturation; below 10 in Fig. S6B) extends the alternative states region and broadens the consumer persistence range across the temperature axis. Note that when the consumer body size ratio is equal to 1 in Communities I and II, the competing species/stages are dynamically identical and the patterns of their persistence and dominance is driven solely by the size–temperature interaction assumed for the resources (i.e., different temperature sensitivities of their R_{max}).

In Community III with smaller relative differences between consumer size classes than in the original model formulation (1, 5 and 10 µg; Fig. S7A), the pattern of species and stages dominance largely resembles the original model (compare to Fig. 4 in the main text). The only substantial difference is that with these alternative body sizes, it is the small consumer biomass that first gets dominated by adults before the same happens in the large consumer (compare the position of orange and yellow dashed lines in Fig. S7A and Fig. 4). When we assumed larger relative differences between consumer size classes than in the original model formulation (0.001, 1 and 10 µg), a diversity of regions of biomass persistence, dominance and bistability opens up in the temperature–diet preference space (Fig. S7B). However, the general pattern remains the same as in the original model (compare with Fig. 4 in the main text) – with increasing temperature, the persistence changes from only the large consumer present to consumer coexistence to only the

small consumer present, and at the same time the stage biomass dominance changes from juveniles to adults in both consumer species (Fig. S7B). Note that regions of alternative stable states occur mostly at moderate to high temperatures, especially for lower values of the diet preference parameter p (i.e., at low levels of intraspecific competition as in Community II; compare Fig. S7B to Fig. 2A in the main text).

We also run the same analysis for all models in which we varied body size ratios while keeping the temperature optima constant (i.e., not as functions of body size) at 24, 20 and 16 °C for the three size classes as in the original model formulation. In this scenario, the patterns of persistence, biomass dominance and alternative stable states were very similar to the original model, and nearly independent from changes in body size ratios. It confirms that body size itself may not be sufficient to produce the expected size shifts with warming as long as it does not modify temperature-dependent processes (in this case, temperature optimum of the maximum ingestion rate; compare with Appendix S2: Fig. S4A and C).

References

Andersen, K. H., T. Berge, R.J. Gonçalves, M. Hartvig, J. Heuschele, S. Hylander, N.S. Jacobsen, C. Lindemann, E.A. Martens, A.B. Neuheimer, K. Olsson, A. Palacz, A.E.F. Prowe, J. Sainmont, S.J. Traving, A.W. Visser, N. Wadhwa, and T. Kiørboe. 2016. Characteristic sizes of life in the oceans, from bacteria to whales. Annual Review of Marine Science 8:217–241.

Angilletta, M. J. Jr., T. D. Steury, and M. W. Sears. 2004. Temperature, growth rate, and body size in ectotherms: fitting pieces of a life-history puzzle. Integrative and Comparative Biology 44:498–509.

Bernhardt, J. R., J. M. Sunday, and M. I. O’Connor. 2018. Metabolic theory and the temperature-size rule explain the temperature dependence of population carrying capacity. American Naturalist 192:687–697.

Brown, J. H., J. F. Gillooly, A. P. Allen, V. M. Savage, and G. B. West. 2004. Toward a metabolic theory of ecology. Ecology 85:1771–1789

Daufresne, M., K. Lengfellner, and U. Sommer. 2009. Global warming benefits the small in aquatic ecosystems. Proceedings of the National Academy of Sciences of the United States of America 106:12788–12793.

Guill, C. 2009. Alternative dynamical states in stage-structured consumer populations. Theoretical Population Biology 76:168–178.

Ikeda, T., Y. Kanno, K. Ozaki, and A. Shinada. 2001. Metabolic rates of epipelagic marine copepods as a function of body mass and temperature. Marine Biology 139:587–596.

Lindmark, M., M. Huss, J. Ohlberger, and A. Gårdmark. 2018. Temperature-dependent body size effects determine population responses to climate warming. Ecology Letters 21:181–189.

Lindmark, M., J. Ohlberger, and A. Gårdmark. 2021. Optimum growth temperature declines with body size within fish species. bioRxiv preprint, doi: https://doi.org/10.1101/2021.01.21.427580.

Nilsson, K. A., L. Persson, and T. Van Kooten. 2010. Complete compensation in *Daphnia* fecundity and stage-specific biomass in response to size-independent mortality. Journal of Animal Ecology 79:871–878.

de Roos, A. M., T. Schellekens, T. van Kooten, K. van de Wolfshaar, D. Claessen, and L. Persson. 2007. Food-dependent growth leads to overcompensation in stage-specific biomass when mortality increases: the influence of maturation versus reproduction regulation. American Naturalist 170:E59–E76.

Savage, V. M., J. F. Gillooly, J. H. Brown, G. B. West, and E. L. Charnov. 2004. Effects of body size and temperature on population growth. American Naturalist 163:429–441.

Uszko, W., S. Diehl, N. Pitsch, K. Lengfellner, and T. Müller. 2015. When is a type III functional response stabilizing? Theory and practice of predicting plankton dynamics under enrichment. Ecology 96:3243–3256.

Uszko, W., S. Diehl, G. Englund, and P. Amarasekare. 2017. Effects of warming on predator–prey interactions: a resource-based approach and a theoretical synthesis. Ecology Letters 20:513–523.

Van Kooten, T., Am. M. de Roos, and L. Persson. 2005. Bistability and an Allee effect as emergent consequences of stage-specific predation. Journal of Theoretical Biology 237:67–74.

Winder, M., J. E. Reuter, and G. S. Schladow. 2008. Lake warming favours small-sized planktonic diatom species. Proceedings of the Royal Society B 276:427–435.

Yvon-Durocher, G., J. M. Montoya, M. Trimmer, and G. Woodward. 2011. Warming alters the size spectrum and shifts the distribution of biomass in freshwater ecosystems. Global Change Biology 17:1681–1694.

Figure S1. Consumer persistence (solid black lines), biomass dominance (dashed black lines) and alternative stable states (solid red lines) boundaries in temperature–background mortality (equal for all consumers within each community, i.e. $\mu_{C_S} = \mu_{C_L}$ or $\mu_J = \mu_A$) space.
A: Two unstructured consumer species feeding on two resources (Community I); B: one stage-structured consumer species feeding on two resources (Community II). In A, on the left hand side of the dashed line, community biomass is dominated by the large consumer C_L, and on the right hand side by the small consumer C_S. In both panels, a size–temperature interaction is present both in the maximum resource density R_{max} and in the temperature optimum of the maximum consumer ingestion rate I_{max}, and the diet preference $p = 0.85$. All equilibria are stable.

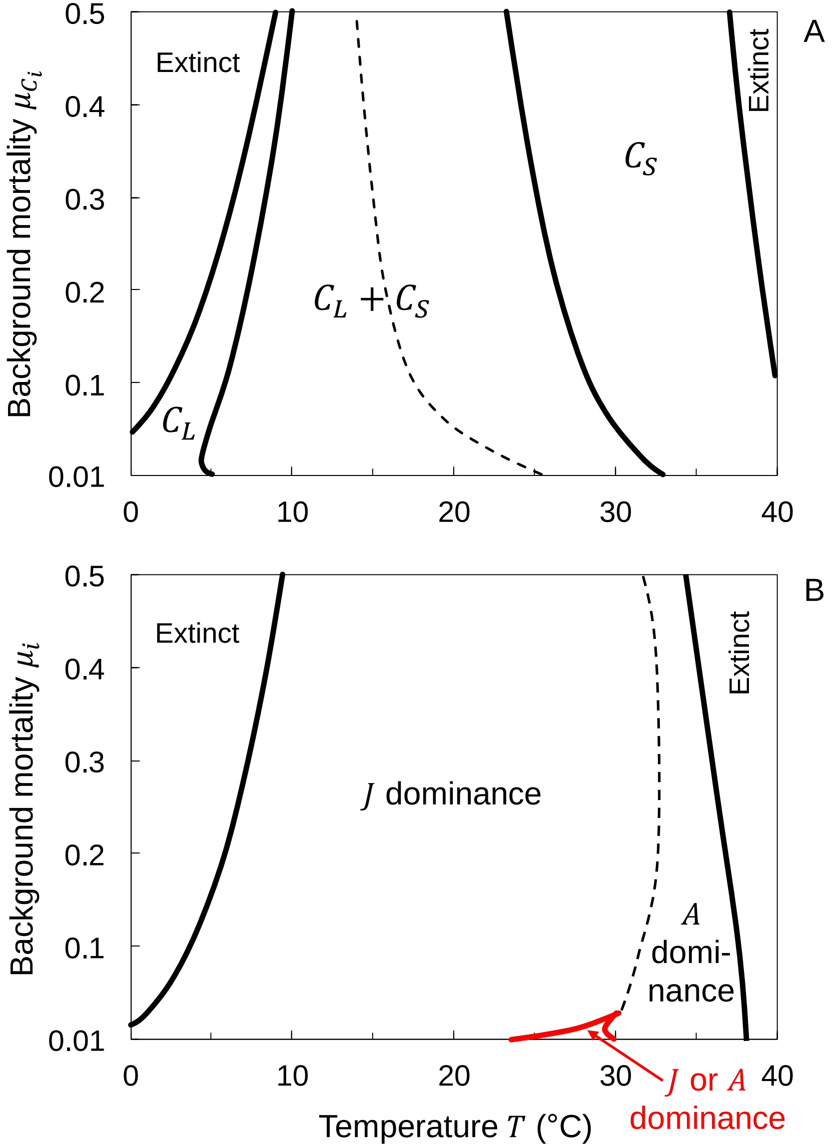

Figure S2. Consumer persistence (solid black lines), biomass dominance (dashed black lines) and alternative stable states (solid red lines) boundaries in temperature–resource enrichment space. Resource enrichment is represented by the coefficient R_0, which scales the default formula of temperature dependence of the maximum (supply) resource biomass densities R_{max} (see Appendix S1: Table S2) of both resources (R_S and R_L).
A: Two unstructured consumer species feeding on two resources (Community I); B: one stage-structured consumer species feeding on two resources (Community II). In A, on the left hand side of the dashed line, community biomass is dominated by the large consumer C_L, and on the right hand side by the small consumer C_S. In both panels, a size–temperature interaction is present both in the maximum resource density R_{max} and in the temperature optimum of the maximum consumer ingestion rate I_{max}, and the diet preference $p = 0.85$. All equilibria are stable.

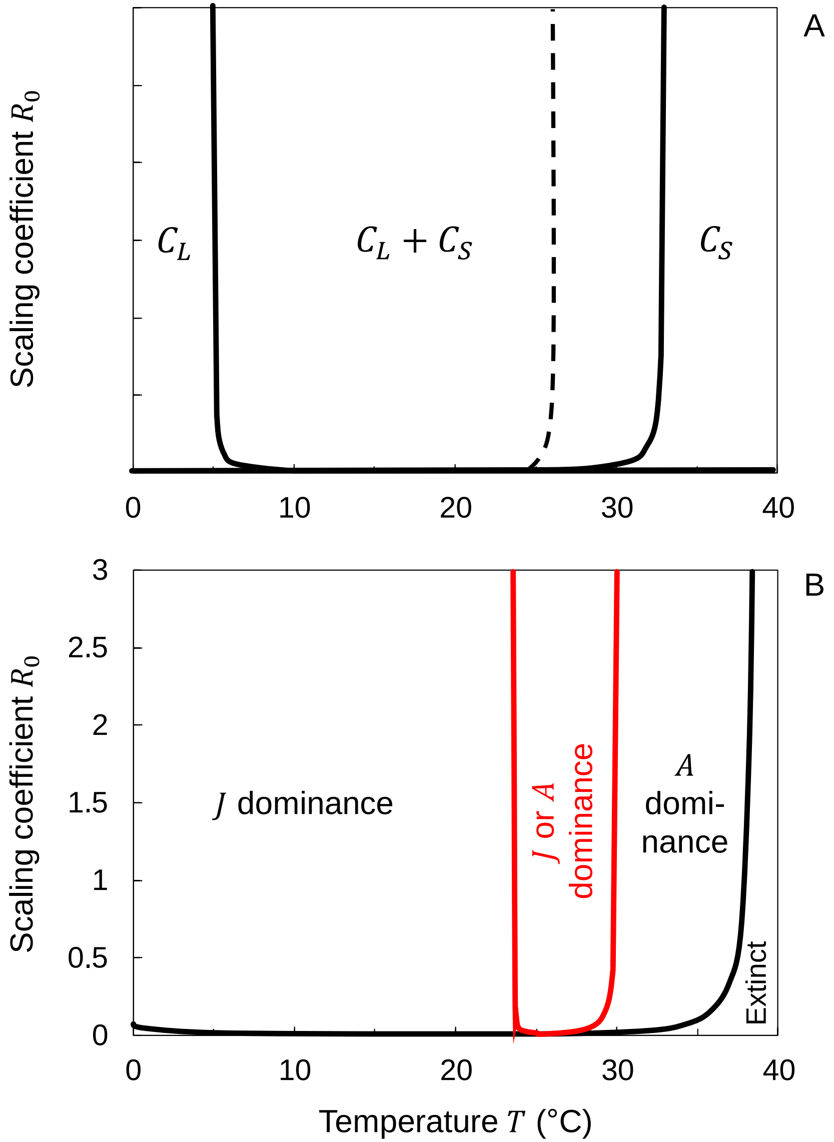

Figure S3. Equilibrium mean individual dry body mass of consumers along the temperature gradient. Left panels (A, B) show model results with no size–temperature interaction. Right panels (C, D) show model results with a size–temperature interaction present both in the maximum resource density R_{max} and in the temperature optimum of the maximum consumer ingestion rate I_{max}. Upper panels (A, C) represent Community I, and lower panels (B, D) represent Community II. Red lines in panel D show an alternative stable state for equilibrium mean individual body mass. In all panels, the diet preference $p = 0.85$.

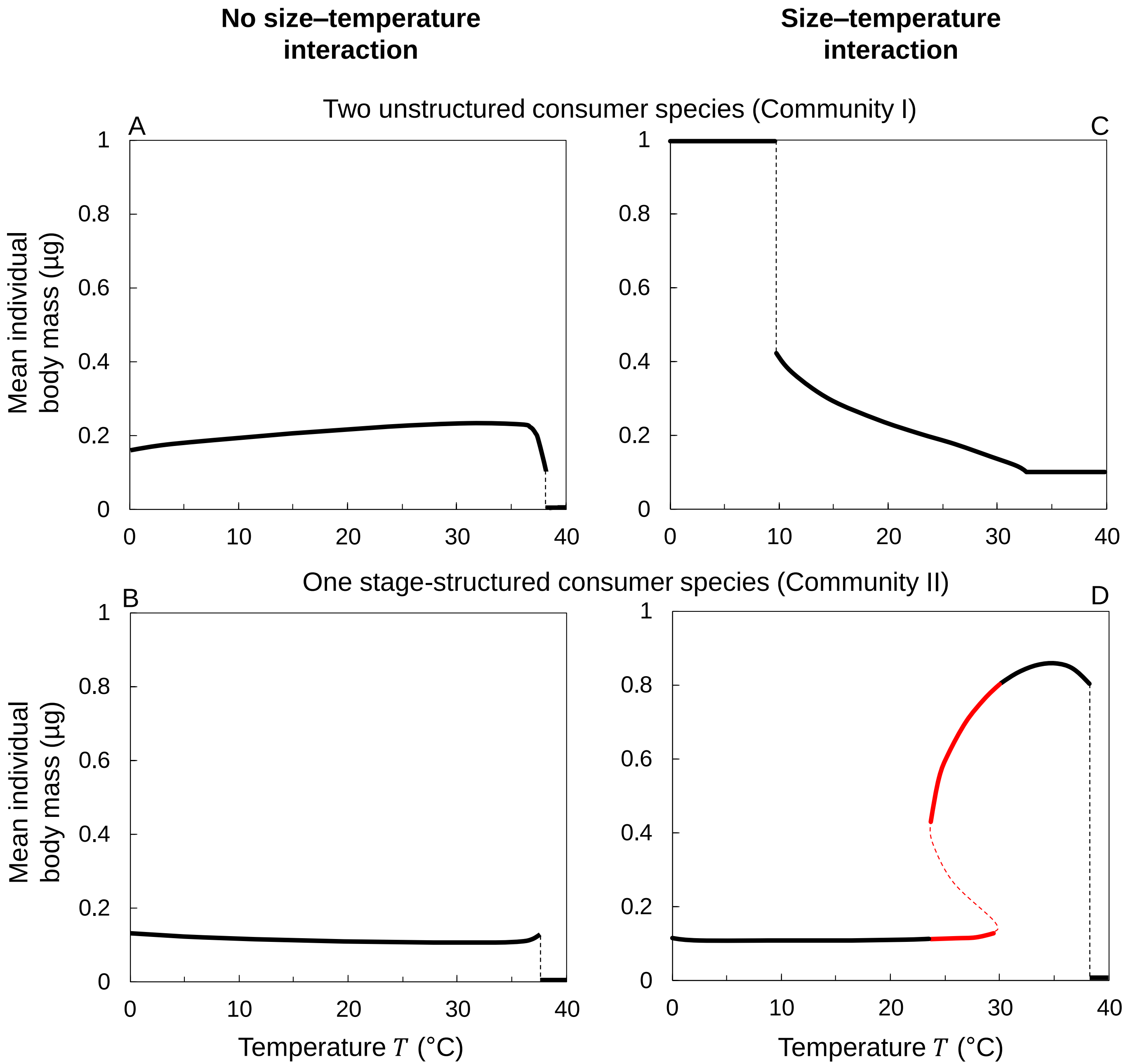

Figure S4. Consumer persistence (solid black lines), biomass dominance (dashed black lines) and alternative stable states (solid red lines) boundaries in temperature–diet preference space. Shown are results for all combinations of the following assumptions on an interaction between body size and temperature: with a size–temperature interaction in the maximum resource density R_{max} either absent ($R_{S\,max}(T) = R_{L\,max}(T)$, panels A, E, I, C, G, K) or present ($R_{S\,max}(T) \neq R_{L\,max}(T)$, panels B, F, J, D, H, L), with a size–temperature interaction in the allometric exponent of the metabolic rate m either present (panels E-H) or absent (all other panels), and with a size–temperature interaction in the temperature optimum of the maximum consumer ingestion rate I_{max} either present (panels I-L) or absent (all other panels). All equilibria are stable. For parameter and rate definitions and formulas, see Appendix S1: Table S2 and Figure S1.

Results are shown for Communities I (two unstructured consumer species feeding on two resources, panels A, B, E, F, I, J) and II (one stage-structured consumer species feeding on two resources, panels C, D, G, H, K, L) Two unstructured consumer species feeding on two resources (community I); B: one stage-structured consumer species feeding on two resources (community II). Note that panels J and L are identical with panels A and B of Fig. 2 in the main text.

Figure S4.

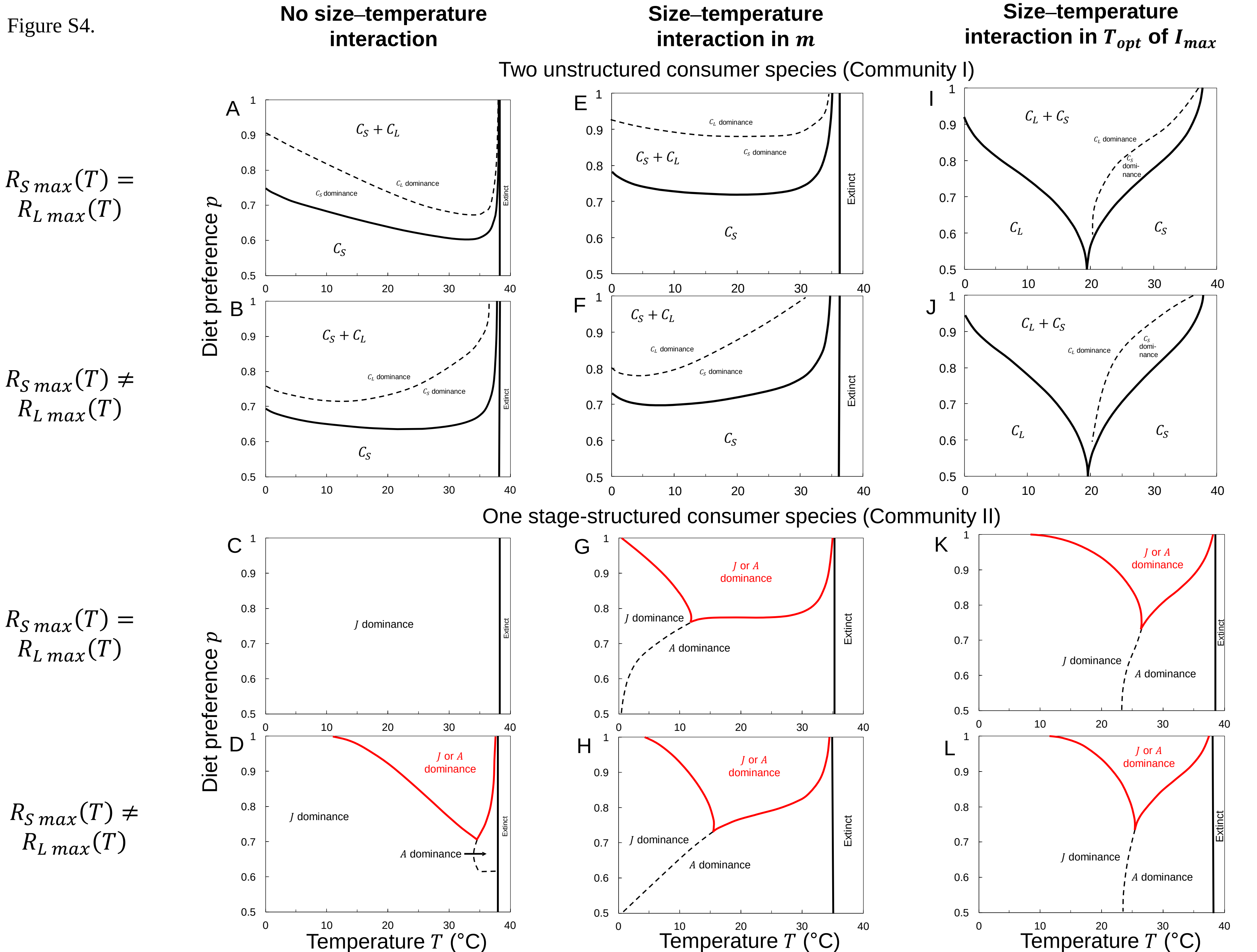
No size–temperature interaction
Size–temperature interaction in m
Size–temperature interaction in T_{opt} of I_{max}
Two unstructured consumer species (Community I)
One stage-structured consumer species (Community II)
$R_{S\,max}(T) = R_{L\,max}(T)$
$R_{S\,max}(T) \neq R_{L\,max}(T)$
Diet preference p
Temperature T (°C)
A
B
C
D
E
F
G
H
I
J
K
L
$C_S + C_L$
$C_L + C_S$
C_S
C_L
C_L dominance
C_S dominance
Extinct
J dominance
A dominance
J or A dominance

Figure S5. Equilibrium mean individual dry body mass along the temperature gradient in Community III (two stage-structured consumer species feeding on two resources); a size–temperature interaction present both in the maximum resource density R_{max} and in the temperature optimum of the maximum consumer ingestion rate I_{max}, and the diet preference $p = 0.75$. Colored regions indicate the presence of only the large consumer (red), both consumers (coexistence; orange), or only the small consumer (yellow), as in Fig. 4 in the main text.

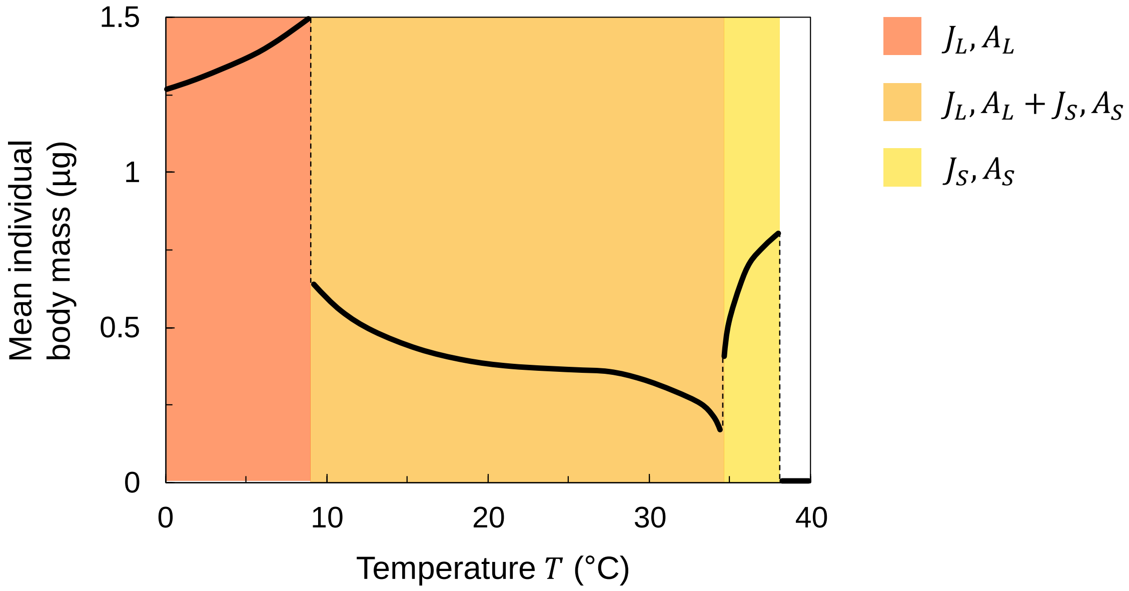

Figure S6. Consumer persistence (solid black lines), biomass dominance (dashed black line) and alternative stable states (solid red lines) boundaries in body mass ratio–temperature space.

A: Two unstructured consumer species feeding on two resources (Community I); B: one stage-structured consumer species feeding on two resources (Community II). In A, below the dashed line community biomass is dominated by the large consumer C_L, and above by the small consumer C_S. In both panels, a size–temperature interaction is present both in the maximum resource density R_{max} and in the temperature optimum of the maximum consumer ingestion rate I_{max} described by $T_{opt} = 305.38 \cdot M^{-0.006}$ (see details in Appendix S2), and the diet preference $p = 0.85$. In B, the consumer persistence boundary is marked with a solid black line. All equilibria are stable. Note the logarithmic x-axes.

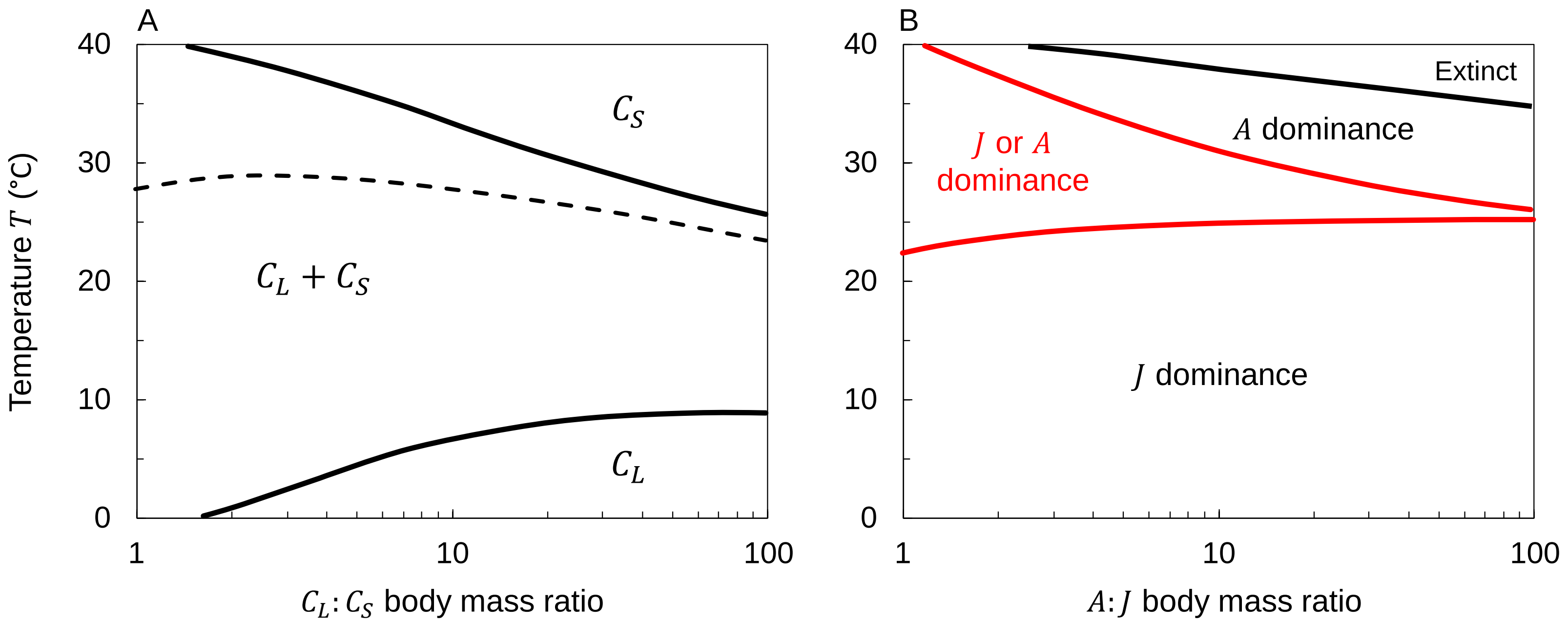

Figure S7. Consumer persistence, biomass dominance and alternative stable states boundaries in temperature–diet preference space in Community III (two stage-structured consumer species feeding on two resources); a size–temperature interaction present both in the maximum resource density R_{max} and in the temperature optimum of the maximum consumer ingestion rate I_{max} described by $T_{opt} = 305.38 \cdot M^{-0.006}$ (see details in Appendix S2).

A: Consumer persistence regions (see the legend for color codes) and biomass dominance boundaries (dashed lines of respective colors) in temperature–diet preference space, assuming 1, 5, and 10 µg dry mass for J_S, A_S and J_L, and A_L, respectively. On the left hand side of the dashed lines, community biomass is dominated by juveniles J_i, and on the right hand side by adults A_i.

B: Consumer persistence regions (the small consumer persists in the region to the right hand side of the yellow solid line, and the large consumer persists in the region to the left hand side of the solid orange line), and biomass dominance and alternative stable states boundaries (black dashed lines; dominance patterns described separately for each region) in temperature–diet preference space, assuming 0.001, 0.1, and 10 µg dry mass for J_S, A_S and J_L, and A_L, respectively. All equilibria are stable.

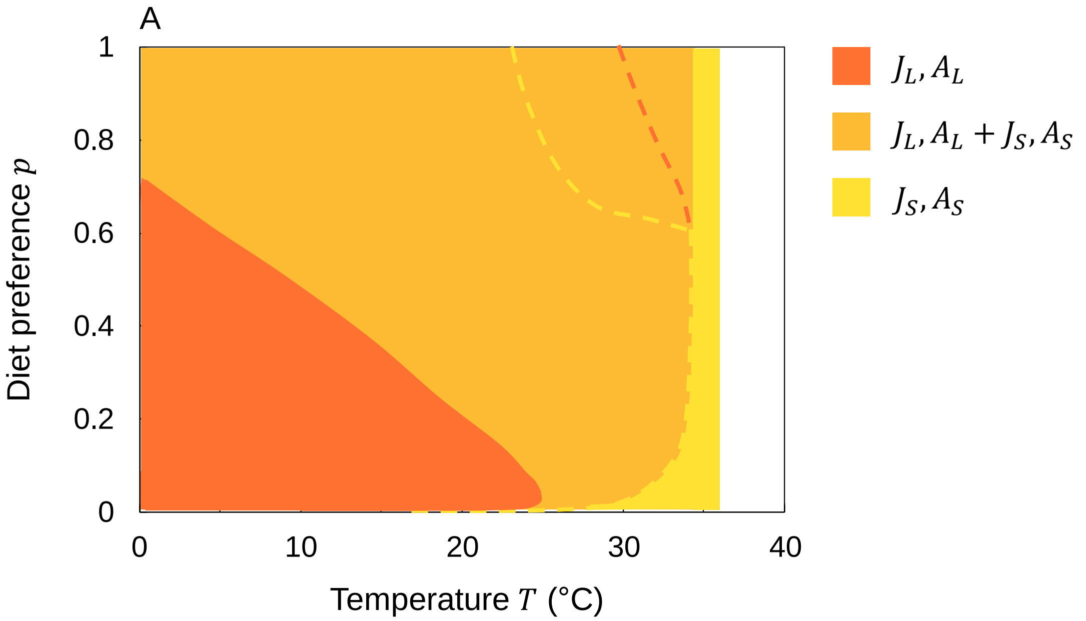

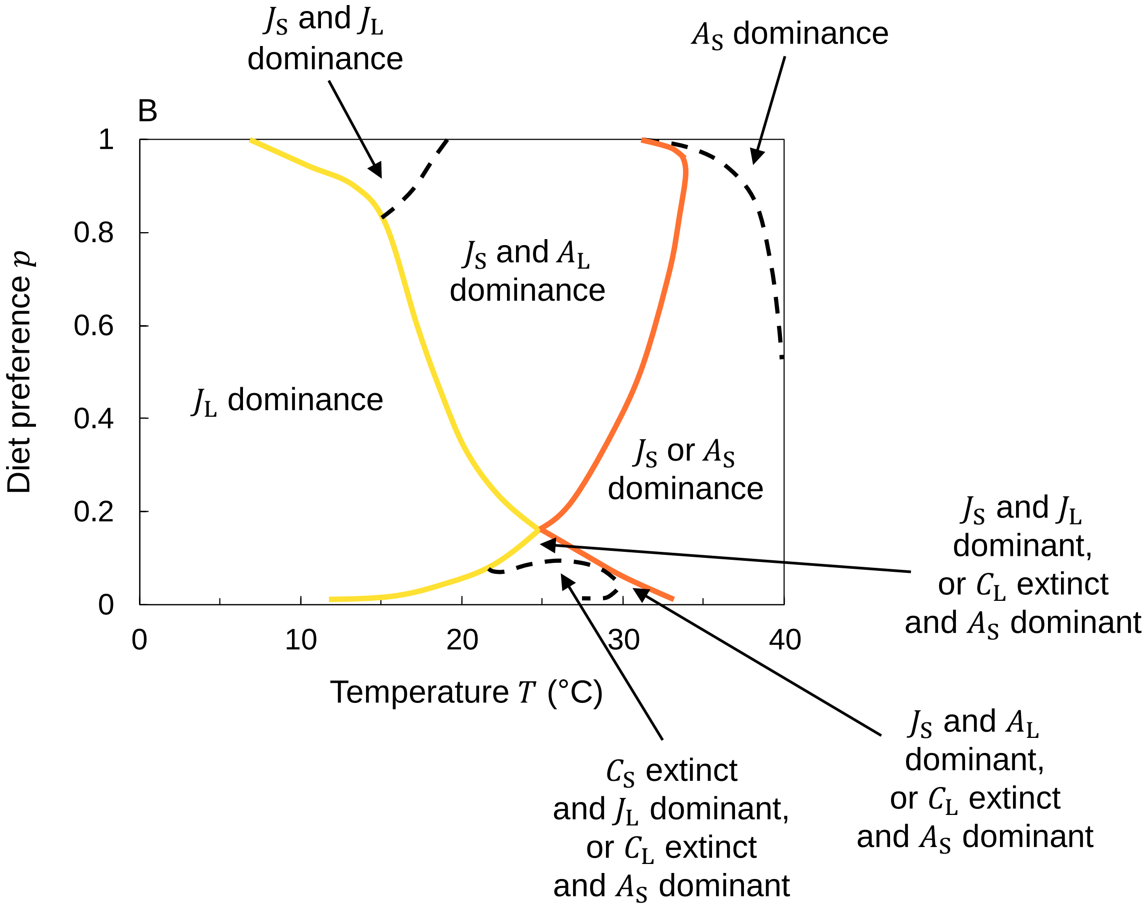

J_S and J_L dominance
A_S dominance
B
Diet preference p
1
0.8
0.6
0.4
0.2
0
J_S and A_L dominance
J_L dominance
J_S or A_S dominance
J_S and J_L dominant, or C_L extinct and A_S dominant
0
10
20
30
40
Temperature T (°C)
C_S extinct and J_L dominant, or C_L extinct and A_S dominant
J_S and A_L dominant, or C_L extinct and A_S dominant
