# Supplementary material for: Smaller species but larger stages: Warming effects on inter‐ and intraspecific community size structure
Source: Ecology. 2022 May 23;103(7):e3699. doi: 10.1002/ecy.3699 (PMC9285768; doi:10.1002/ecy.3699)
Supplement: Supplementary file 3 — Appendix S3 [file ECY-103-0-s001.pdf]

Journal name: Ecology

Manuscript title: Smaller species but larger stages: Warming effects on inter- and intraspecific community size structure

Authors: Wojciech Uszko, Magnus Huss (ORCID ID 0000-0002-5131-6000) and Anna Gårdmark (ORCID ID 0000-0003-1803-0622)

Corresponding author: Wojciech Uszko, email address: wojciech.uszko@protonmail.com

Affiliation: Department of Aquatic Resources, Swedish University of Agricultural Sciences, Skolgatan 6, SE-742 42, Öregrund, Sweden

Appendix S3: Review of the literature on warming-induced changes in intra- and interspecific size structure of competitive zooplankton communities.

Overview

The purpose of this review was to collect and qualitatively summarize results from published studies on temperature-driven changes in size structure of competitive zooplankton communities, both within and between species.

Shifts in population or community body size can occur at three different levels (Daufresne et al. 2009, Ohlberger at al. 2013): (1) change in individual size (size-at-age shift), (2) change in juvenile vs. adult proportion (population structure shift), and (3) change in species proportions (species composition shift). The mechanisms responsible for changes in level (1) are tackled by the so-called temperature-size rule (Atkinson 1994, Ohlberger 2013) and are not dealt with in this study. Instead, we focus on levels (2) and (3), and try to

find qualitative patterns in the existing literature on how warming influences the stage (intraspecific) and species (interspecific) structure of zooplankton grazers. We focus on exploitation (resource) competition – that is, relative competitive ability of species and stages – as the main factor shaping community size structure.

Our aim was not to exhaustively and quantitatively find, analyse and describe all existing literature studies on the subject. Instead, our purpose was to get a solid qualitative overview of the relevant results present in ecological literature.

Methods

We have searched the published literature (years 1945 – May 2020) using Web of Science, with search words *zooplankton* AND (*temperature* OR *warming*) AND (*size* OR *juvenile* OR *nauplii* OR *adult*). After the initial search, the total number of potentially relevant articles reached > 540.

For a study to be included in the final list, the following conditions had to be met (assessed by screening entire articles):

1. Zooplankton as the study subject. Specifically, we were interested in all protist and metazoan heterotrophs, spanning the size from single-celled protists to oceanic salps, krill and jellyfish, from every kind of aquatic pelagic environment. The majority of the studies in the final list focused on crustacean zooplankton.

2. Zooplankton consumers had to be engaged in resource competition. We included only studies in which plankton consumers inhabited the same experimental or natural area, so that they can compete for shared resources. For instance, studies that looked at zooplankton

performance across temperature for different species (stages) kept in separate experimental tanks were not included.

3. Body size of zooplankton individuals needed to be explicitly reported, either in units of length or weight, or in relative terms (i.e. smaller/larger). We found several studies that describe changes in zooplankton communities with warming, but without explicit reporting of species body sizes or stage structure. These studies were excluded.

4. Temperature change as the factor (the only one, or one of several that were studied) behind size structure changes. We included all studies describing the effects of at least two ambient/experimental temperatures. We assumed that temperature drives the changes in vital rates and life history parameters (even if they have not been explicitly identified), thus influencing relative competitive ability of zooplankton species and stages. However, in many cases, temperature was not the only factor that could potentially influence zooplankton size structure. Other factors included, for instance, food availability, nutrient or light enrichment, predation pressure, physical factors, etc.

5. We included both experimental and observational studies. An experimental study is defined as one that involves a manipulation of either study subjects or conditions (i.e., treatments). This includes laboratory and mesocosm experiments (indoors and outdoors/in situ), and intentional lake manipulations. An observational study is defined as one that does not manipulate the study subjects and conditions, but instead passively observes them. This includes lake and sea sampling/monitoring (including monitoring of areas that were anthropogenically altered for purposes other than research, for instance cooling reservoirs of power plants), across-latitude/geographical area comparisons, as well as studies using subfossil material.

6. We included studies that span all potential spatial scales (i.e., from microcosm to mesocosm to field studies) as well as temporal scales (few days to weeks/months to studies across one or many seasons/years).

From each study in the final list of articles, we noted which of the following three types of observations a given study had made: (1) shift from larger to smaller stages/species with warming, (2) shift from smaller to larger stages/species with warming, and (3) no significant effect observed. Note that one study (i.e., article) can make more than one observation. We divided all observations into four groups, which are combinations of experimental vs. observational studies at the intraspecific (stage or clone structure) vs. interspecific (species composition) level.

Results

In total, the final list included 136 studies (articles) that deal with warming effects on competitive zooplankton communities and report responses in body size. The full list of studies, together with short descriptions of observed effects, is found in the reference section at the end of this Appendix. These studies have reported 164 observations. Fig. 5 in the main text contains a summary of all observations across the four categories. The detailed list of studies which report given observations is found in Table S1 below. For detailed description of results, see the main text (Discussion).

We would like to note that the observed effects in some cases could not be clearly identified as driven solely by temperature. For instance, in short mesocosm studies, observations might be the result of specific initial conditions and transient dynamics, e.g. an increase in juvenile abundance if an experiment has been inoculated with adult individuals only. Other examples include potential effects of other factors, such as fish predation

pressure, food quality limitation, or presence of cyanobacteria (see the reference list description below where we comment on all such ambiguous cases).

References

Atkinson, D. 1994. Temperature and organism size—A biological law for ectotherms? Advances in Ecological Research 25:1–58.

Daufresne, M., K. Lengfellner, and U. Sommer. 2009. Global warming benefits the small in aquatic ecosystems. Proceedings of the National Academy of Sciences of the United States of America 106:12788–12793.

Ohlberger, J. 2013. Climate warming and ectotherm body size - from individual physiology to community ecology. Functional Ecology 27:991–1001.

Table S1. The published 136 literature studies included in the review, divided into the considered categories, and identified by a number between 1 and 136 as listed in the reference list below. Note that a few numbers (articles) appear in more than one category if they reported more than a single observation.

	Observation	Studies reporting the observation at **intraspecific level**	Studies reporting the observation at **interspecific level**
Experimental studies	Shift to **smaller** stages/species with warming	16, 17, 48, 49, 116	7, 19, 34, 43, 44, 45, 49, 51, 62, 64, 72, 82, 84, 86, 90, 100, 111, 113, 117, 121, 124
	Shift to **larger** stages/species with warming	32, 65, 109, 113, 121, 124, 135	1, 2, 32, 79, 108, 135
	No effect	55, 110	50, 59, 90, 95, 110, 122, 132, 133, 136
Observational studies	Shift to **smaller** stages/species with warming	3, 6, 33, 36, 37, 46, 75, 77, 96, 104, 105, 118, 120, 129, 134	4, 5, 8, 9, 10, 11, 12, 13, 14, 15, 18, 19, 20, 21, 22, 24, 25, 26, 27, 28, 29, 30, 33, 39, 40, 41, 42, 46, 47, 52, 53, 57, 58, 60, 61, 63, 67, 68, 70, 71, 73, 75, 76, 78, 81, 83, 86, 88, 89, 91, 92, 93, 94, 97, 99, 101, 102, 106, 107, 112, 114, 115, 123, 125, 126, 127, 128, 130, 131, 134
	Shift to **larger** stages/species with warming	26, 35, 36, 37, 66, 80, 89, 96, 98, 130	21, 25, 38, 54, 60, 69, 85, 103
	No effect	69, 119	11, 23, 31, 56, 70, 74, 77, 87, 119

Reference list with short result summaries

1. Aberle, N., Lengfellner, K. & Sommer, U. (2007). Spring bloom succession, grazing impact and herbivore selectivity of ciliate communities in response to winter warming. *Oecologia*, 150, 668–681.

Large ciliates dominate later in the season and in warmer treatment in Kiel indoor mesocosms.

2. Aberle, N., Bauer, B., Lewandowska, A, Gaedke, U. & Sommer, U. (2012). Warming induces shifts in microzooplankton phenology and reduces time-lags between phytoplankton and protozoan production. *Mar. Biol.*, 159, 2441–2453

Ciliate extinction after peaking transiently with warming; copepods survive because they peak later; almost 2-month-long experiment.

3. Adamczuk, M. (2012). Spatial distribution of juvenile and adult stages of limnetic Cladocera in relation to selected environmental factors. *J. Limnol.*, 71, 112–118.

One season monitoring of Lake Piaseczno, Poland, April-November. Positive correlation between temperature and juvenile abundance of five species of Cladocera; no effect on adults.

4. Adrian, R. & Deneke, R. (1996). Possible impact of mild winters on zooplankton succession in eutrophic lakes of the Atlantic European area. *Freshwater Biol.*, 36, 757–770.

Shift from larger *Daphnia galeata* to smaller *Daphnia cucullata* in Heiligensee (close to Berlin) over 19 years of data.

5. Alheit, J. & Niquen, M. (2004). Regime shifts in the Humboldt Current ecosystem. *Prog. Oceanogr.*, 60, 201–222.

Decrease in abundance of large copepods in warmer periods in Humboldt Current system offshore Peru, and its connection to anchovy-sardine dynamics.

6. Ambriz-Arreola, I., Gómez-Gutiérrez, J., del Carmen Franco-Gordo, M.,. Lavaniegos. B.E. & Godínez-Domínguez, E. (2012). Influence of coastal upwelling−downwelling variability on tropical euphausiid abundance and community structure in the inshore Mexican central Pacific. *Mar. Ecol. Prog. Ser.*, 451, 119–136.
West offshore Mexico, observations on euphasiids before, during, and after the El Nino event 1996-1998. Larval abundance higher in warmer El Nino waters, and later stages (juvenile and adults) more abundant in colder water.

7. Atkinson, D., Ciotti, B.J. & Montagnes, D.J.S. (2003). Protists decrease in size linearly with temperature: *ca*. 2.5% °C^{-1}. *Proc. R. Soc. Lond. B*, 270, 2605–2611
Decrease in protist size across many studies, 2.5% per 1 °C.

8. Balayla, D., Lauridsen, T.L., Søndergaard, M. & Jeppesen, E. (2010). Larger zooplankton in Danish lakes after cold winters: are winter fish kills of importance? *Hydrobiologia*, 646, 159–172.
Observations made in 37 Danish lakes across a few years, from which 1996 had a particularly

cold winter, which resulted in higher proportion of larger taxa in summer. Proposed main explanation involves higher fish mortality during colder winter.

9. Balazy, K., Trudnowska, E., Wichorowski, M. & Błachowiak-Samołyk, K. (2018). Large versus small zooplankton in relation to temperature in the Arctic shelf region. *Polar Res.*, 37, 1427409.
Larger size fraction was more abundant in colder years in West Spitsbergen Shelf 2010-2016. Note that the smaller size fraction consisted not only of smaller species, but also nauplii, obscuring the effect.

10. Batchelder, H.P., Daly, K.L., Davis, C.S., Ji, R., Ohman, M.D., Peterson, W.T. & Runge, J.A. (2013). Climate impacts on zooplankton population dynamics in coastal marine ecosystems. *Oceanography*, 26, 34–51.
Shift to smaller copepods during warm periods of Pacific Decadal Oscillation in the California current system.

11. Batten, S.D., Moffitt, S., Pegau, W.S. & Campbell, R. (2016). Plankton indices explain interannual variability in Prince William Sound herring first year growth. *Fish. Oceanogr.*, 25, 420–432.
Change in timing but not in abundance of small and large copepods between cold and warm years. However, increase in microzooplankton in warmer years. Alaskan Gulf, 2000-2013.

12. Batten, S.D., Raitsos, D.E., Danielson, S., Hopcroft, R., Coyle, K. & McQuatters-Gollop, A. (2018). Interannual variability in lower trophic levels on the Alaskan Shelf. *Deep-Sea Res. Pt II*, 147, 58–68.
Shift to smaller copepods both between and within years when warmer, on the Alaskan Shelf 2000-2003.

13. Beaugrand, G., Brander, K.M., Lindley, J.A., Souissi, S. & Reid, P.C (2003). Plankton effect on cod recruitment in the North Sea. *Nature*, 426, 661–664.
Decrease in mean calanoid copepod size in the North Sea during 1960-2000.

14. Beaugrand, G. Decadal changes in climate and ecosystems in the North Atlantic Ocean and adjacent seas. (2009). *Deep-Sea Res. Pt II*, 56, 656–673.
Shift to smaller calanoid copepods in North Atlantic with higher surface temperature over decades.

15. Beaver, J.R., Tausz, C.E., Renicker, T.R., Holdren, G.C., Hosler, D.M., Manis, E.E., Scotese, K.C., Teacher, C.E., Vitanye, B.T. & Davidson, R.M. (2014). The late summer crustacean zooplankton in western U.S.A reservoirs reflects ecoregion, temperature and latitude. *Freshwater Biol.*, 59, 1173–1186.
Late summer 2010 across 102 western US reservoirs. More of smaller species in warmer waters and in lower latitudes.

16. Beisner, B., McCauley, E. & Wrona, F.J. (1996). Temperature-mediated dynamics of planktonic food chains: the effect of an invertebrate carnivore. *Freshwater Biol.*, 35, 219–232.
Transient dominance of *Mesostoma* juveniles before extinction in 3-month-long mesocosm experiment.

17. Beisner, B., McCauley, E. & Wrona, F.J. (1997). The influence of temperature and food chain length on plankton predator-prey dynamics. *Can. J. Fish. Aquat. Sci.*, 54, 586–595.
Less adult-dominated transient before *Daphnia* went extinct due to juveniles failing to mature.

18. Bengtsson, J. (1987). Competitive dominance among Cladocera: Are single-factor explanations enough? In: Forró L., Frey D.G. (eds) Cladocera. Developments in Hydrobiology, vol 35. Springer, Dordrecht.
Shift to smaller *Daphnia* species at higher water temperature in rock pools.

19. Bernot, R.J., Dodds, W.K., Quist, M.C. et al. (2006). Temperature and kairomone induced life history plasticity in coexisting *Daphnia*. *Aquat. Ecol.*, 40, 361–372.
Shift with warming in kairomone presence from larger *Daphnia pulicaria* to smaller *Daphnia mendotae*. Shift through one season in Glen Elder reservoir from *Daphnia pulicaria* to *Daphnia mendotae*, coinciding with warming but also with stronger fish predation.

20. Bjerring, R., Becares, E., Declerck, S., Gross, E.M., Hansson, L.-A., Kairesalo, T., Nykänen, M., Halkiewicz, A., Kornijów, R., Conde-Porcuna, J.M., Seferlis, M., Noges, T., Moss, B., Amsinck, S.L., Odgaard, B.V. & Jeppesen, E. (2009). Subfossil Cladocera in relation to contemporary environmental variables in 54 Pan-European lakes. *Freshwater Biol.*, 54, 2401–2417.

Smaller species associated with lower latitudes and warmer lakes, vice versa for larger Cladocera species, in subfossil, sediment material of 54 lakes across Europe.

21. Brucet, S., Boix, D., Gascon, S., Sala, J., Quintana, X.D., Badosa, A., Søndergaard, M., Lauridsen, T.L & Jeppesen, E. (2009). Species richness of crustacean zooplankton and trophic structure of brackish lagoons in contrasting climate zones: north temperate Denmark and Mediterranean Catalonia (Spain). *Ecography*, 32, 692–702.

35 and 42 brackish lagoons in Spain and Denmark, respectively. Shift from copepod to rotifer dominance comparing winter vs. summer in Spain, but vice versa in Denmark. Differences in salinity as a confounding factor.

22. Brucet, S., Boix, D., Quintana, X.D., Jensen, E., Nathansen, L.W., Trochine, C., Meerhoff, M., Gascon, S. & Jeppesen, E. (2010). Factors influencing zooplankton size structure at contrasting temperatures in coastal shallow lakes: Implications for effects of climate change. *Limnol. Oceanogr.*, 55, 1697–1711.

Shift to smaller species comparing four Danish with four Spanish lakes; in the latter no larger size classes present, no cladocerans, and rotifer dominance.

23. Carter, J.L. & Schindler, D.E. (2012). Responses of zooplankton populations to four decades of climate warming in lakes of Southwestern Alaska. *Ecosystems*, 15, 1010–1026.
No change in summer densities of all dominant zooplankton taxa from 1963 to 2009, despite increase in summer temperatures in Alaskan lakes. Increase in cladoceran production though.

24. Chiba, S., Tadokoro, K., Sugisaki, H. & Ino, T. (2006). Effects of decadal climate change on zooplankton over the last 50 years in the western subarctic North Pacific. *Glob. Change Biol.*,12, 907–920.
Decline in spring-summer large copepod abundance with warming in northwest subarctic Pacific.

25. Chiba, S., Batten, S.D., Yoshiki, T., Sasaki, Y., Sasaoka, K., Sugisaki, H. & Ichikawa, T. (2015). Temperature and zooplankton size structure: climate control and basin-scale comparison in the North Pacific. *Ecol Evol.*, 5, 968–978.
Decline in temperature optimum (=temperature at abundance peak) with body size in northwest Pacific copepods. However, due to particularities of temperature isoclines, more of larger species in warmer waters.

26. Cooney, R.T., Coyle, K.O., Stockmar, E. & Stark, C. (2001). Seasonality in surface-layer net zooplankton communities in Prince William Sound, Alaska. *Fish. Oceanogr.*, 10, 97–109.
Adults and late copepodite stages dominated during summer in Alaskan gulf over the season, but no strong patterns present; seasonality of reproduction plays an important role. Larger copepods dominant only in winter and spring, the rest of the season smaller ones dominate.

27. Coyle, K.O., Pinchuk, A.I., Eisner, L.B. & Napp, J.M. (2008). Zooplankton species composition, abundance and biomass on the eastern Bering Sea shelf during summer: The potential role of water-column stability and nutrients in structuring the zooplankton community. *Deep-Sea Res. Pt II*, 55, 1775– 1791.
Strong shift from large to small zooplankton (mostly copepods) from 1990s to 2000s in the Barents Sea.

28. Cremona, F., Agasild, H., Haberman, J., Zingel, P., Nõges, P., Nõges, T. & Laas, A. (2020). How warming and other stressors affect zooplankton abundance, biomass and community composition in shallow eutrophic lakes. *Climatic Change*, 159, 565–580.
General increase in biomass of ciliates, rotifers and cladocerans, and decrease in copepods, during 38 years in shallow Estonian lake. Small-bodied cyclopoids increase though.

29. Dalpadado, P., Ingvaldsen, R. & Hassel, A. (2003). Zooplankton biomass variation in relation to climatic conditions in the Barents Sea. *Polar Biol.*, 26, 233–241.
Increase in small bodied zooplankton in warm vs. cold year in the Barents Sea, 1981-2000.

30. Debertin, A.J., Hanson, J.M. & Courtenay, S.C. (2018). Linking zooplankton assemblages with oceanographic zones in an Atlantic coastal ecosystem. *Can. J. Fish. Aq. Sci.*, 75,868–882.
Dominance of smaller-bodied species such (copepods, *Thecosomata*, crab zoeae and hermit

crab larvae) in warmer water, and larger-bodied (*Calanus* copepods, chaetognaths and *Podon sp.*) in colder waters of Canadian shore waters.

31. Dimas-Flore, N., Alcocer, J. & Ciros-Pérez, J. (2008). The structure of the zooplankton assemblages from two neighboring tropical high mountain lakes. *J. Freshwater Ecol.*, 23, 21–31.
No clear seasonal trend in zooplankton size structure in two lakes in Mexico. Rotifers abundant in the fall, copepods fluctuating through the year, cladocerans generally declining after spring peak.

32. van Doorslaer, W., Stoks, R., Swillen, I., Feuchtmayr, H., Atkinson, D., Moss, B. & De Meester, L. (2010). Experimental thermal microevolution in community-embedded *Daphnia* populations. *Clim. Res.*, 43, 81–89.
Short mesocosm experiment (20 and 24 °C) showed more of larger *Daphnia magna* than smaller *Daphnia pulex*. At the same time, after 6 months of adaptation, clone structure shifted to larger ones in warmer conditions, especially in *Daphnia pulex*.

33. Du, P., Jiang, Z.B., Zhu, Y.L. et al. What factors control the variations in abundance, biomass, and size of mesozooplankton in a subtropical eutrophic bay? (2020). *Estuaries Coasts*, 43, 2128–2140.
Full year 2015 in Xiangshan Bay, China. Warmer waters are associated with higher abundance of small-bodied copepods, but also more nauplii.

34. Dupuis, A.P. & Hann, B.J. (2009). Warm spring and summer water temperatures in small eutrophic lakes of the Canadian prairies: potential implications for phytoplankton and zooplankton. *J Plankton Res.*, 31, 489–502.
Two consecutive springs (colder then warmer) in Canadian shallow lakes; decrease in *Daphnia* species, increase in rotifers, as well as increase of one copepod species and *Bosmina*.

35. Durbin, E.G., Garrahan, P.R. & Casas, M.C. (2000). Abundance and distribution of *Calanus finmarchicus* on the Georges Bank during 1995 and 1996. *ICES J Mar. Sci.*, 57, 1664–1685.
Less nauplii and more later stages of copepods when temperature is warmer May-June, in the Georges Bank, NW Atlantic, 1995-1996.

36. Dutz, J., Mohrholz, V. & van Beusekom, J.E.E. (2010). Life cycle and spring phenology of *Temora longicornis* in the Baltic Sea. *Mar. Ecol. Prog. Ser.*, 406, 223–238.
Bornholm Basin of the Baltic Sea, 2002-2003. Strong seasonal signal in the life cycle of the copepod, with overwintering adults, nauplii peaking few times per season, and summer dominated mostly by older stages.

37. Dvoretsky, V.G. & Dvoretsky, A.G. (2009). Life cycle of *Oithona similis* (Copepoda: Cyclopoida) in Kola Bay (Barents Sea). *Mar. Biol.*, 156, 1433–1446.
Strong seasonal signal in the life cycle of the copepod. Nauplii peak in early summer, then they strongly decline and copepodites are stably abundant when it is still warm, and then

overwinter with little higher densities than adults, the latter peaking in early autumn when temperature is still high, especially lower in the water column.

38. Dvoretsky, V.G. & Dvoretsky, A.G. (2013). Epiplankton in the Barents sea: Summer variations of mesozooplankton biomass, community structure and diversity. *Cont. Shelf Res.*, 52, 1–11.
Exceptionally warm years (around 2006) brought more herbivorous and large-bodied zooplankton in the Barents Sea between 2003 and 2009.

39. Dzierzbicka-Glowacka, L., Żmijewska, I.M., Mudrak, S., Jakacki, J. & Lemieszek, A. (2010). Population modelling of *Acartia* spp. in a water column ecosystem model for the South-Eastern Baltic Sea. *Biogeosciences*, 7, 2247–2259.
Experimental data from year 2000 in Gdansk Gulf show that in summer, smaller-bodied taxa dominate (*Bosmina*, rotifers), unlike in winter with mostly copepods.

40. Escribano, R., Daneri, G., Farías, L., Gallardo, V. et al. (2004). Biological and chemical consequences of the 1997–1998 El Niño in the Chilean coastal upwelling system: a synthesis. *Deep-Sea Res. Pt II*, 51, 2389–2411.
Strong El Nino event 1997-1998 caused a shift towards warmer waters, and more abundant smaller zooplankton species, offshore Chile and Peru.

41. Evans, E.L., Hirst, A.G., Kratina, P. & Beaugrand, G. (2019). Temperature-mediated changes in zooplankton body size: large scale temporal and spatial analysis. *Ecography*, 00,

1–10, doi: 10.1111/ecog.04631

Shift to smaller copepod species in North Atlantic at lower latitudes and higher temperatures.

42. Evans R., Lea, M.-A., Hindell, M.A. & Swadling, K.M. (2020). Significant shifts in coastal zooplankton populations through the 2015/16 Tasman Sea marine heatwave. *Estuar. Coast. Shelf S.*, 235, 106538.

Shift towards smaller species, and towards larvae (of nonplanktonic organisms, therefore functionally smaller species here), when warmer offshore Tasmania 2015-2018.

43. Feniova I.Y., Razlutsky, V. I. & Palash, A.L. (2011). Temperature effects of interspecies competition between cladoceran species in experimental conditions. *Inland Water Biol.*, 4, 65–71.

Shift from larger (*Daphnia magna* and *Simocephalus*) to smaller (*Diaphanosoma* and *Ceriodaphnia*) competing zooplankton species with warming in the lab (18 to 25 °C).

44. Fey, S.B. & Cottingham, K.L. (2011). Linking biotic interactions and climate change to the success of exotic *Daphnia lumholtzi*. *Freshwater Biol.*, 56, 2196–2209.

Competitive dominance of *Daphnia lumholtzi* over *Daphnia pulex* with warming.

45 Fey, S.B. & Cottingham, K.L. (2012). Thermal sensitivity predicts the establishment success of nonnative species in a mesocosm warming experiment. *Ecology*, 93, 2313–2320.

Competitive dominance of smaller invasive *Daphnia lumholtzi* over native larger *Daphnia pulex* in heated mesocosms.

46. Florencia Gutierre, M., Devercelli, M., Brucet, S., Lauridsen, T.L, Søndergaard, M. & Jeppesen, E. (2016). Is recovery of large-bodied zooplankton after nutrient loading reduction hampered by climate warming? A long-term study of shallow hypertrophic Lake Søbygaard, Denmark. *Water*, 8, 341.
During 23 years of nutrient loading recovery of shallow lake Søbygaard, Denmark, small-bodied species increased in abundance, however nauplii (yearly average) built up more and more proportion of all copepods.

47. Gao, X., Chen, H., Govaert, L., Wang, W &, Yang, J. (2019). Responses of zooplankton body size and community trophic structure to temperature change in a subtropical reservoir. *Ecol Evol.*, 9, 12544–12555.
Shift to smaller species, mostly due to taxonomic sorting along increasing temperature in a subtropical reservoir in China.

48. Garzke, J., Ismar, S.M.H. & Sommer, U. (2015). Climate change affects low trophic level marine consumers: warming decreases copepod size and abundance. *Oecologia*, 177, 849–860.
Shift from copepodite to nauplii domination; 28 day mesocosm experiment; it could have been driven by transient hatching and mortality differences.

49. Garzke, J., Hansen, T., Ismar, S.M.H. & Sommer, U. (2016). Combined effects of ocean warming and acidification on copepod abundance, body size and fatty acid content. *PLoS*

ONE, 11, e0155952.
Over 25 days of mesocosm experiment, in the warm treatment (15 vs. 9 °C) smaller species were more dominant (*Oithona sp.*), and abundances of all copepod stages declined, but it was strongest in adults.

50. Garzke, J., Connor, S.J., Sommer, U. & O'Connor, M.I. (2019). Trophic interactions modify the temperature dependence of community biomass and ecosystem function. *PLoS Biol.*, 17, e2006806.
No effects on zooplankton body size (average, all species considered) after 9 weeks of mesocosm experiment in Vancouver, BC, Canada, with temperature gradient 19-30 °C.

51. Gauthier, J., Prairie, Y.T. & Beisner, B.E. (2014). Thermocline deepening and mixing alter zooplankton phenology, biomass and body size in a whole-lake experiment. *Freshwater Biol.*, 59, 998–1011.
Whole-lake manipulation (Lac Croche, Canada), mixing the waters and deepening the thermocline. This caused higher proportion of smaller species, and the mean zooplankton body size declined significantly compared to the control basin.

52. Gerten, D. & Adrian, R. (2002). Species-specific changes in the phenology and peak abundance of freshwater copepods in response to warm summers. *Freshwater Biol.*, 47, 2163–2173.
Among three considered copepods species in Müggelsee, Germany, 1980-1999, the largest one increased in abundance the least in the warmer years.

53. Gillooly, J.F. & Dodson, S.I. (2000). Latitudinal patterns in the size distribution and seasonal dynamics of new world, freshwater cladocerans. *Limnol. Oceanogr.*, 45, 22–30.
Shift to smaller species with decreasing latitude (=increasing temperature) through shifts in species composition in 1100 water bodies in the Americas.

54. González-Gil, R., González Taboada, F., Höfer, J. & Anadón, R. (2015). Winter mixing and coastal upwelling drive long-term changes in zooplankton in the Bay of Biscay (1993–2010). *J Plankton Res.*, 37, 337–351.
Increase in abundance of larger zooplankton size classes when warmer in the Cantabrian Sea, Bay of Biscay, 1993-2010. Potentially the role of upwelling and mixing as the cause.

55. Gusha, M.N.C., Dalu, T., Wasserman, R.J. & McQuaid, C.D. (2019). Zooplankton grazing pressure is insufficient for primary producer control under elevated warming and nutrient levels. *Sci. Total Environ.*, 651, 410–418.
Microcosm experiment at 17 and 24 °C. No clear change in adult vs. naupliar copepod abundance with temperature across all nutrient addition treatments.

56. Gyllström, M., Hansson, L.-A., Jeppesen, E., Criado, F., García Gross, E., Irvine, K., Kairesalo, T., Kornijow, R., Miracle, M.R., Nykänen, M., Nõges, T., Romo, S., Stephen, D., Donk, E. & Van Moss, B. (2005). The role of climate in shaping zooplankton communities of shallow lakes. *Limnol. Oceanogr.*, 6, doi: 10.4319/lo.2005.50.6.2008.

No effect of temperature in zooplankton size of shallow lake communities across Europe; definitely less *Daphnia* in warm though.

57. Hart Rob C. (2004). Cladoceran periodicity patterns in relation to selected environmental factors in two cascading warm-water reservoirs over a decade. *Hydrobiologia*, 526, 99–117. South Africa, two warm reservoirs, 10 years of observations. Larger daphnids are present the entire year, and in summer come smaller species. Generally, temperature affects the abundance of the former negatively, and of the latter positively.

58. Haven, K.E., Pinto-Coelho, R.M., Beklioglu, M., Christoffersen, K.S. et al. (2015). Temperature effects on body size of freshwater crustacean zooplankton from Greenland to the tropics. *Hydrobiologia*, 743, 27–35

Shift to smaller cladocerans and cyclopoids, but not calanoids, with warming across 122 lakes from eight different regions from Greenland to the tropics.

59. Hu, H., Jin, H., Jeppesen, E., Li, K., Liu, Z. & Zhang, Y. (2018). Fish-mediated plankton responses to increased temperature in subtropical aquatic mesocosm ecosystems: Implications for lake management. *Water Res.*, 144, 304e311.

Four-months-long mesocosm experiment, China. No decline in proportion of large daphnids in warmed mesocosms, unless fish were present.

60 Hillbricht-Ilkowska, A. & Zdanowski, B. (1978). Effect of thermal effluents and retention time on lake functioning and ecological efficiencies in plankton communities. *Int. Revue ges.*

Hydrobiol. Hydrogr., 63, 609–617.
Shift to smaller *Daphnia* (from *D. longispina* to *D. cucullata*), but also decrease in rotifers, in lakes used for power plant cooling near Konin, Poland.

61. Hillbricht-Ilkowska, A., Ejsmont-Karabin, J. & Weglenska, T. (1988). Long-term changes in the composition, productivity and trophic efficiency in the zooplankton community of heated lakes near Konin (Poland). *Ekol. Pol.*, 36, 115–144.
Three artificially warmed lakes in Poland; shift to smaller species (mostly rotifers), decrease in cladocerans.

62. Holzapfel, A. & Vinebrooke, R.D. (2005). Environmental warming increases invasion potential of alpine lake communities by imported species. *Glob. Change Biol.*, 11, 2009–2015.
Shift to smaller, invasive species of Alpine ponds in warmed treatment in the lab; 13 and 20 °C.

63. Hopcroft, R.R., Roff, J.C. & Chavez, F.P. (2001). Size paradigms in copepod communities: a re-examination. *Hydrobiologia*, 453/454, 133–141.
Size spectra of copepods reveal that in temperate waters there are more of larger species compared to tropical waters, even though small species dominate everywhere.

64. Horn, H.G., Boersma, M., Garzke, J. et al. (2020). High CO2 and warming affect microzooplankton food web dynamics in a Baltic Sea summer plankton community. *Mar.*

Biol., 167, 69.

Mesocosm experiment in the Baltic Sea, 16.5 and 22.5 °C and different CO_2 concentration levels. Less copepods, more ciliates in warmer treatments.

65. Hu, S.S. & Tessier, A.J. (1995). Seasonal succession and the strength of intra- and interspecific competition in a *Daphnia* assemblage. *Ecology*, 76, 2278–2294.

There were generally more adults in the warmer part of the season in mesocosms (lake enclosures) in *Daphnia galeata mendotae* in Gull Lake, Michigan.

66. Hülsmann, S. & Voigt, H. (2002). Life history of *Daphnia galeata* in a hypertrophic reservoir and consequences of non-consumptive mortality for the initiation of a midsummer decline. *Freshwater Biol.*, 47, 2313–2324.

Increase in proportion of adults in *Daphnia* towards summer in Bautzen Reservoir, Germany, 1998.

67. Huo, Y., Sun, S., Zhang, F., Wang, M., Li, C. & Yang, B. (2012). Biomass and estimated production properties of size-fractionated zooplankton in the Yellow Sea, China. *J. Mar. Sys.*, 94, 1–8.

Yellow Sea, China, 2006-2007. The largest zooplankton body size fraction, unlike other fractions, does not peak in summer, and those other, smaller groups decline in winter.

68. Iglesias, C., Mazzeo, N., Meerhoff, M. et al. (2011). High predation is of key importance for dominance of small-bodied zooplankton in warm shallow lakes: evidence from lakes, fish

exclosures and surface sediments. *Hydrobiologia*, 667, 133–147.
Seasonal shift from larger cladocerans to rotifers from winter to summer, in two subtropical Uruguayan lakes.

69. Taticchi, M.I., Pandolfi, P., Biondi, M.T. & Sebastiani, P. (1992). Population dynamics of *Daphnia galeata* Sars and *Bosmina longirostris* Muell in Lake Trasimeno, Italy. *Ital. J Zool.*, 59, 427–435.
No clear effect on stage abundance in both studied species in Lake Trasimeno, Italy, 1989. However, the larger *Daphnia* was always dominant in abundance, yet more so in summer months.

70. Jansson, M., Jonsson, A., Andersson, A. & Karlsson, J. (2010). Biomass and structure of planktonic communities along an air temperature gradient in subarctic Sweden. *Freshwater Biol.*, 55, 691–700.
Across 15 lakes of different latitudes, and therefore water temperature, in subarctic Sweden, there is no clear pattern in zooplankton size structure, as for instance fish presence has stronger effect. However, when looking at fishless lakes only, proportion of ciliates and rotifers increases with temperature.

71. Jensen, T.C. (2019). Winter decrease of zooplankton abundance and biomass in subalpine oligotrophic Lake Atnsjøen (SE Norway). *J. Limnol.*, 78, 34–363.
Rotifers dominating the total zooplankton abundance and biomass, but only in the growing seasons, of 2010-2011.

72. Johnsen M.A., Stuparyk, B.R., Cook, J. & Vinebrooke, R. (2020). Resilience of pond communities to extreme thermal regime shifts: an alpine–montane reciprocal transplant experiment. *Aq. Sci.*, 82, 38.
Shift towards smaller species when warmer in alpine and montane lakes where communities were reciprocally transplanted; Canada.

73. Johnson, C.R., Banks, S.C., Barrett, N.S. et al. (2011). Shifts in oceanography, species' ranges and subtidal marine community dynamics in eastern Tasmania. *J. Exp. Mar. Biol. Ecol.*, 400, 17–32.
Ocean around Tasmania, decline in big krill in warm years, shift to ‘warm’ zooplankton species but no size mentioned.

74. Jones, N.T. & Gilbert, B. (2018). Geographic signatures in species turnover: decoupling colonization and extinction across a latitudinal gradient. *Oikos*, 127, 507–517.
Temperature was not a predictor of changes in zooplankton community structure across 43 West Canada lakes. Smaller species indeed increased, but rather due to their higher colonization success.

75. Kang, Y., Kimb, S. & Lee, W. (2006). Seasonal and spatial variations of zooplankton in the central and southeastern Bering Sea during the mid-1990s. *Deep-Sea Res. Pt I*, 53, 795–803.

More of copepodites and less of adults in summer and fall, but probably due to reproduction cycle rather than intraspecific competition. Seasonal shift to smaller taxa.

76. Kelly, P., Clementson, L., Davies, C., Corney, S. & Swadling, K. (2016). Zooplankton responses to increasing sea surface temperatures in the southeastern Australia global marine hotspot. *Est. Coast. Shelf Sci.*, 180, 242e257.
Shift towards smaller copepods in warming water in SE Australia.

77. Kimmel, D.G., Eisner, L.B., Wilson, M.T. & Duffy-Anderson, J.T. (2018). Copepod dynamics across warm and cold periods in the eastern Bering Sea: Implications for walleye pollock (*Gadus chalcogrammus*) and the Oscillating Control Hypothesis. *Fish Oceanogr.*, 27, 143–158.
No change in abundance of smaller species warm vs. cold, however an increase in larger-sized stages of *Calanus* in colder periods; SE Bering Sea 2001-2011.

78. Kraft, A., Nöthig, E.-M., Bauerfeind, E. et al. (2013). First evidence of reproductive success in a southern invader indicates possible community shifts among Arctic zooplankton. *Mar. Ecol. Prog. Ser.*, 493, 291–296.
Spread of small amphipod *Themisto* towards Artic waters around Svalbard.

79. Kratina, P., Greig, H.S., Thompson, P.L., Carvalho-Pereira, T.S.A. & Shurin, J.B. (2012). Warming modifies trophic cascades and eutrophication in experimental freshwater

communities. *Ecology*, 93, 1421–1430.
Shift to larger species in winter under warming.

80. Kvile, K.Ø., Dalpadado, P., Orlova, E., Stenseth, N.C. & Stige, L.C. (2014). Temperature effects on *Calanus finmarchicus* vary in space, time and between developmental stages. *Mar. Ecol. Prog. Ser.*, 517, 85–104.
Increasing temperatures (mean anomalies, and seasonal) lead to lower abundances of nauplii, and higher of late copepodites and adults, in the Barents Sea, 1959-1992.

81. Lacerot, G., Paggi, J.C., de Paggi, S.J. et al. (2010). Fish size explains zooplankton size along a latitudinal gradient in South America. In: Effects of climate on size structure and functioning of aquatic food webs. Thesis, Wageningen University, Wageningen, NL.
Decrease in body size of zooplankton communities across 81 shallow lakes in South America (5-55°S).

82. Lewandowska, A.M., Boyce, D.G., Hofmann, M., Matthiessen, B., Sommer, U. & Worm, B. (2014). Effects of sea surface warming on marine plankton. *Ecol. Lett.*, 17, 614–623.
Less copepods and more ciliates in warm marine mesocosms; shift to grazing on ciliates in copepods.

83. Li, Y., Xie, P., Zhao, D., Zhu, T., Guo, L. & Zhang, J. (2016). Eutrophication strengthens the response of zooplankton to temperature changes in a high-altitude lake. *Ecol. Evol.*, 6, 6690–6701.

Shift towards higher proportion of smaller species – *Bosmina* and rotifers – in Lake Erhai, China, in summer. Otherwise larger *Daphnia* dominant.

84. Lydersen, E., Aanes, K.J., Andersen, S. et al. (2008). Ecosystem effects of thermal manipulation of a whole lake, Lake Breisjøen, southern Norway (THERMOS project). Hydrology and Earth System Sciences Discussions, European Geosciences Union, 12, 509–522.
Less cladocerans, more of small-bodied copepods after artificial mixing treatment resulting in higher temperatures in Lake Breisjøen, southern Norway.

85. MacLennan, M.M., Arnott, S.E. & Strecker, A.L. (2012). Differential sensitivity of planktonic trophic levels to extreme summer temperatures in boreal lakes. *Hydrobiologia*, 680, 11–23.
Larger species in higher proportion in anomalously warm summer 2005 compared to the normal one 2006, in 20 boreal lakes in Canada.

86. Maclennan, M.M., Dings-Avery, C. & Vinebrooke, R.D. (2015). Invasive trout increase the climatic sensitivity of zooplankton communities in naturally fishless lakes. *Freshwater Biol.*, 60, 1502–1513.
Shift to smaller species in mesocosm (+3.8 °C). Shift to smaller species in both fish-absent and fish-present lakes in Canada.

87. MacLennan, M.M. & Vinebrooke, R.D. (2016). Effects of non-native trout, higher temperatures and regional biodiversity on zooplankton communities of alpine lakes. *Hydrobiologia*, 770, 193–208.
No clear effect of warming on zooplankton size structure, as both large as well as small herbivores were similarly negatively affected, with no relative changes in proportions.

88. Makinen, K., Vuorinen, I. & Hanninen, J. (2017). Climate-induced hydrography change favours small-bodied zooplankton in a coastal ecosystem. *Hydrobiologia*, 792, 83–96.
Decline in large-, increase in small-bodied zooplankton in north Baltic Sea archipelago between 1967-2013, due to temperature increase but also salinity decline.

89. Marrari, M., Daly, K.L, Timonin, A. & Semenova, T. (2011). The zooplankton of Marguerite Bay, Western Antarctic Peninsula — Part I: Abundance, distribution, and population response to variability in environmental conditions. *Deep-Sea Res. Pt II*, 58, 1599–1613.
More adults in warmer 2001 than in colder 2002 in Marguerite Bay, Antarctic. More of smaller taxa (pteropods, smaller copepods) in 2001 than 2002.

McKee, D., Atkinson, D., Collings, S., Eaton, J., Harvey, I., Heyes, T., Hatton, K., Wilson, D. & Moss, B. (2002), Macro-zooplankter responses to simulated climate warming in experimental freshwater microcosms. *Freshwater Biol.*, 47, 1557–1570.
Very small effect of 2-year warming in microcosms; no substantial changes in community size spectrum; tendency towards smaller cladoceran and ostracod species though.

91 Moline, M.A., Claustre, H., Frazer, T.K., Schofield, O. ^ Vernet, M. (2004). Alteration of the food web along the Antarctic Peninsula in response to a regional warming trend. *Glob. Change Biol.*, 10, 1973–1980.

Antarctic Peninsula, 1991-1996: warming-induced shift from diatoms to smaller cryptophytes, therefore shift from krill to salps.

92. Molinero, J.C., Anneville, O., Souissi, S., Balvay, G. & Gerdeaux, D. (2006). Anthropogenic and climate forcing on the long-term changes of planktonic rotifers in Lake Geneva, Europe, *J. Plankton Res.*, 28, Pages 287–296.

Shift to smaller rotifers in Lake Geneva during 1970-1998.

93. Nevalainen, L., Ketola, M., Korosi, J.B. et al. (2014). Zooplankton (Cladocera) species turnover and long-term decline of *Daphnia* in two high mountain lakes in the Austrian Alps. *Hydrobiologia*, 722, 75–91.

Increase in small *Bosmina*, decrease in larger *Daphnia*, when temperature increases long-term in two Austian alpine lakes.

94. Nicolas, D., Rochette, S., Llope, M &, Licandro, P. (2014). Spatio-temporal variability of the North Sea cod recruitment in relation to temperature and zooplankton. *PLoS ONE*, 9, e88447.

Increase in small (< 2 mm) zooplankton species in the North Sea, 1974-2011, concurrent with warmer sea surface temperature.

95. O'Connor, M.I., Piehler, M.F., Leech, D.M., Anton, A. & Bruno, J.F. (2009). Warming and resource availability shift food web structure and metabolism. *PLoS Biol.*, 7, e1000178.
Outdoor microcosms, from ambient to +6 °C; no effect on zooplankton length.

96. Persson, J., Stige, L.C., Stenseth, N.C., Usov, N. & Martynova, D. (2012). Scale-dependent effects of climate on two copepod species, *Calanus glacialis* and *Pseudocalanus minutus*, in an Arctic-boreal sea. *Mar. Ecol. Prog. Ser.*, 468, 71–83.
White Sea, >50 years of observations, with concurrent warming. In *Calanus*, the relative proportion of nauplii increased, and in *Pseudocalanus* decreased, with time and warming.

97. Pitois, S.G. & Fox, C.J. (2006). Long-term changes in zooplankton biomass concentration and mean size over the Northwest European shelf inferred from Continuous Plankton Recorder data. *ICES J. Mar. Sci.*, 63, 785e798.
Decline in copepod body size coinciding with temperature increase in North Atlantic and the North Sea.

98. Pretorius, M., Huggett, J.A. & Gibbons, M.J. (2016). Summer and winter differences in zooplankton biomass, distribution and size composition in the KwaZulu-Natal Bight, South Africa. *African J. Mar. Sci.*, 38, S155–S168.
Summer copepod community in KwaZulu-Natal Bight, South Africa, 2010, was dominated by larger, older individuals. In cooler winter waters, in contrast, by smaller and younger.

99. Ramos-Jiliberto, R. & Aranguiz-Acuna, A. (2007). Between-species differences in demographic responses to temperature of coexisting cladocerans. *Austral Ecol.*, 32, 766–774.
Less larger species (e.g. *Daphnia ambigua*) and more smaller species (e.g. *Moina micrura*) at higher temperatures in a temperate lake in Chile.

100. Rasconi, S., Gall, A., Winter, K. & Kainz, M.J. (2015). Increasing water temperature triggers dominance of small freshwater plankton. *PLoS ONE*, 10, e0140449.
Larger abundance of smaller species (mostly *Bosmina*) in warm treatments (+3 °C) in a mesocosm experiment.

101. Redmond, L.E., Loewen, C J G. & Vinebrooke, R.D. (2018). A functional approach to zooplankton communities in Mountain Lakes stocked with nonnative sportfish under a changing climate. *Water Resource Res.*, 54, 2362–2375.
Small body size of species associated with higher temperature, and vice versa for larger size, across 137 mountain lakes in Western Canada.

102. Rice, E., Dam, H.G. & Stewart, G. (2015). Impact of climate change on estuarine zooplankton: surface water warming in Long Island Sound is associated with changes in copepod size and community structure. *Est. Coasts*, 38, 13–23.
Shift in zooplankton community away from larger species (e.g. *Metridia* and *Candacia* that completely disappeared) towards smaller ones (e.g. *Oithona*) in the Central Basin of Long Island Sound, from late 1940s, concurrent with increasing temperature.

103. Rose, J.M., Feng, Y., Gobler, C.J., Gutierrez, R., Hare, C.E., Leblanc, K. & Hutchins, D.A. (2009). Effects of increased pCO2 and temperature on the North Atlantic spring bloom. II. Microzooplankton abundance and grazing. *Mar. Ecol. Prog. Ser.*, 388, 27–40.
Microzooplankton (ciliates as the largest group) in North Atlantic – dominance of larger ciliates in warmer and smaller ciliates in colder waters.

104. Saunders, P.A., Porter, K.G. & Taylor, B.E. (1999). Population dynamics of *Daphnia* spp. and implications for trophic interactions in a small, monomictic lake. *J. Plankton Res.*, 21, 1823–1845.
Dominance of adults in winter and juveniles in spring-summer in *Daphnia* in Lake Oglethorpe, Georgia, USA.

105. Schlüter, M.H., Merico, A., Reginatto, M., Boersma, M., Wiltshire, K.H. & Greve, W. (2010). Phenological shifts of three interacting zooplankton groups in relation to climate change. *Glob. Change Biol.*, 16, 3144–3153.
Stronger decline in adults than in juveniles coinciding with sea surface temperature increase in southern North Sea (1974-2005) in ctenophore *Pleurobrachia pileus*.

106. Schmoke, C. & Hernández-León, S. (2013). Stratification effects on the plankton of the subtropical Canary Current. *Prog. Oceanogr.*, 119, 24–31.
Relatively more of smaller than larger mesozooplankton species in summer months 2005-2007, offshore Canary Islands.

107. Selmeczy, G.B., Abonyi, A., Krienitz, L. et al. (2019). Old sins have long shadows: climate change weakens efficiency of trophic coupling of phyto- and zooplankton in a deep oligo-mesotrophic lowland lake (Stechlin, Germany)—a causality analysis. *Hydrobiologia*, 831, 101–117.
Long-term decrease in abundance of larger copepod species, and increase in *Bosmina* and *Diaphnosoma*, in Lake Stechlin, Germany, concurrent with warming 1994-2014.

108. Sikora, A. & Dawidowicz, D. (2014). Do the presence of filamentous cyanobacteria and an elevated temperature favor small-bodied *Daphnia* in interspecific competitive interactions? *Fundam. Appl. Limnol.*, 185, 307–314.
Larger *Daphnia pulicaria* always competitively superior to smaller *Daphnia longispina*, also with warming and in cyanobacteria presence.

109. Sommer, U., Aberle, N. & Engel, A. et al. (2007). An indoor mesocosm system to study the effect of climate change on the late winter and spring succession of Baltic Sea phyto- and zooplankton. *Oecologia*, 150, 655–667.
Faster decline in nauplii when warm, so more of larger individuals in indoor Kiel mesocosms.

110. Šorf, M., Davidson, T.A., Brucet, S. et al. (2015). Zooplankton response to climate warming: a mesocosm experiment at contrasting temperatures and nutrient levels. *Hydrobiologia*, 742, 185–203.
No clear effect of temperature during 3 months of mesocosm experiment. Nonsignificant results for zooplankton taxa as well as stages.

111. Strecker A.L., Cobb, T.P. & Vinebrooke, R.D. (2004). Effects of experimental greenhouse warming on phytoplankton and zooplankton communities in fishless alpine ponds. *Limnol. Oceanogr.*, 49, 1182–1190.
Decline of large cladocerans, no changes in copepods, increase of small rotifers in mesocosms imitating Canadian fishless ponds; warming +3.6 °C.

112. Suikkanen, S., Pulina, S., Engstrom-Ost et al. (2013). Climate change and eutrophication Induced shifts in northern summer plankton communities. *PLoS ONE*, 8, e66475.
Decline in total zooplankton and cladocerans, but increase in rotifers in northern Baltic Sea (Åland Sea) during 1979-2011.

113. Tavsanogl, U.N., Sorf, M., Stefanidis, K. et al. (2017). Effects of nutrient and water level changes on the composition and size structure of zooplankton communities in shallow lakes under different climatic conditions: a pan-European mesocosm experiment. *Aquat. Ecol.*, 51, 257–273
Mesocosms across Europe, Sweden to Greece: less cladocerans, more copepods, lower nauplii-to-total copepods ratio, higher small cladocerans-to-total cladocerans ratio with increasing temperature.

114. Taylor, B.E. & Mahoney, D.L (1988). Extinction and recolonization: Processes regulating zooplankton dynamics in a cooling reservoir. *SIL Proceedings*, 23:3, 1536–1541.
Shift from cladocerans to rotifers in a power plant cooling reservoir.

115. Thompson, G.A., Dinofrio, E.O. &. Alder, V.A. (2012). Interannual fluctuations in copepod abundance and contribution of small forms in the Drake Passage during austral summer. *Helgol. Mar. Res.*, 66, 127–138.

Positive effect of sea surface temperature on the abundance of smaller size class of copepods (< 300 µm), vice versa for the larger size, in the Drake Passage 2000-2003.

116. Thompson, P.L., St-Jacques, M.-C. & Vinebrooke, R.D. (2008). Impacts of climate warming and nitrogen deposition on slpine plankton in lake and pond habitats: an in vitro experiment. *Arctic Antarctic Alpine Res.*, 40, 192–198.

Unclear effect of temperature (8 and 15 °C) on adult vs. juvenile abundance; typically less adults, but not always more juveniles, when warmer.

117. Tunowski, J. (2009). Zooplankton structure in heated lakes with differing thermal regimes and water retention. *Arch. Pol. Fish.*, 17, 291–303.

Dominance of rotifers in heated lakes near Konin power plants, Poland.

118. Turner, J.T. (1982). The annual cycle of zooplankton in a Long Island estuary. *Estuaries*, 5, 261–274.

Adults dominate in winter, nauplii in summer in Long Island estuary, but it might have been caused by seasonality of reproduction rather than the temperature effect. At the same time, it is adults, not juveniles, which overwinter.

119. Turner, J.T., Borkman, D.G. & Libby, P.S. (2011). Zooplankton trends in Massachusetts Bay, USA: 1998–2008. *J. Plankton Res.*, 33, 1066–1080.

No significant effect of temperature on zooplankton species and stages in Massachusetts Bay, USA: 1998–2008.

120. Uye, S., Aoto, I. & Onbé, T. (2002). Seasonal population dynamics and production of *Microsetella norvegica*, a widely distributed but little-studied marine planktonic harpacticoid copepod. *J. Plankton Res.*, 24, 143–153.

As nauplii appear and peak in warm summer months, adult copepods overwinter, temperature decreases relative abundance of adults in the Inland Sea of Japan, 1982-1983.

121. Van de Perre, D., Janssen, C.R. & De Schamphelaere, K.A.C. (2018). Combined effects of interspecies interaction, temperature, and zinc on *Daphnia longispina* population dynamics. *Environ. Toxic. Chem.*, 37, 1668–1678.

Increase in small rotifer abundance relative to *Daphnia* in warm (21-22 °C, compared to cold 17-18 °C) treatments. At the same time, with no competition, *Daphnia* intrapopulation structure shifts towards relatively more adults in the warm treatment after 21 experimental days.

122. Velthuis, M., de Senerpont Domis, L.N., Frenken, T. et al. (2017). Warming advances top-down control and reduces producer biomass in a freshwater plankton community. *Ecosphere*, 8, e01651.

No clear pattern in size-specific zooplankton community structure with warming, as both rotifer and cladoceran abundances increased.

123. Venkataramana, V., Anilkumar, N., Naik, R.K., Mishra, R.K. & Sabu, P. (2019). Temperature and phytoplankton size class biomass drives the zooplankton food web dynamics in the Indian Ocean sector of the Southern Ocean. *Polar Biol.*, 42, 823–829.
Under warm stratified conditions, dominance of small copepods in the Indian part of the Southern Ocean in 2015.

124. Vidussi, F., Mostajir, B., Fouilland, E. et al. (2011). Effects of experimental warming and increased ultraviolet B radiation on the Mediterranean plankton food web. *Limnol. Oceanogr.*, 56, doi: 10.4319/lo.2011.56.1.0206.
After 10 days of mesocosm experiment in Sète, France, there were relatively more adults than nauplii in the warm treatment (+3 °C) than in the control. At the same time, relative abundance of ciliates and heterotrophic flagellates increased there as well.

125. Viñas, M.D., Negri, R.M., Cepeda, G.D. et al. (2013). Seasonal succession of zooplankton in coastal waters of the Argentine Sea (Southwest Atlantic Ocean): prevalence of classical or microbial food webs. *Mar. Biol. Res.*, 9, 371–382.
Relative dominance of small copepods in summer, with more of microbial loop food web, in the Argentine Sea, 2000-2001. In winter, classic herbivore food web with dominance of large copepods.

126. Volkov, A.F. (2012). The results of zooplankton studies in the Bering Sea under the NPAFC Program (BASIS Expedition). Part 1. Eastern Areas. *Russian J. Mar. Biol.*, 38, 474–

494.
Shift from more small species-dominated zooplankton community in the eastern Bering Sea during the warmer period 2003-2006, to larger species in the colder period 2007-2011.

127. Wagner, C. & Adrian, R. (2011). Consequences of changes in thermal regime for plankton diversity and trait composition in a polymictic lake: a matter of temporal scale. *Freshwater Biol.*, 56, 1949–1961.
Warmer, stratified periods led to higher rotifer biomass, and lower cladoceran biomass, in Müggelsee, Germany, 1982-2007.

128. Wang, W., Sun, S., Zhang, F. et al. (2018). Zooplankton community structure, abundance and biovolume in Jiaozhou Bay and the adjacent coastal Yellow Sea during summers of 2005–2012: relationships with increasing water temperature. *J. Ocean. Limnol.*, 36, 1655–1670.
Increase in the abundance of smaller-bodied copepods in Jiaozhou Bay and adjacent Yellow Sea, concurrent with increase in water temperature in Augusts of 2005-2012.

129. Wilson, S.E., Swalethorp, R., Kjellerup, S., Wolverton, M.A., Ducklow, H.W. & Yager, P.L. (2015). Meso- and macro-zooplankton community structure of the Amundsen Sea Polynya, Antarctica (Summer 2010–2011). *Elem. Sci. Anth.*, 3, 000033.
Positive correlation between euphasiid larvae to adult ratio and temperature, in Amundsen Sea Polynya, Antarctica, 2010-2011.

130. Winder, M. & Schindler, D.E. (2004). Climate change uncouples trophic interactions in an aquatic ecosystem. *Ecology*, 85, 2100–2106.

Stronger juvenile than adult decline in Lake Washington over few decades. Shift from cladocerans to rotifers over few decades as mean temperature increases.

131. Yamaoka, H., Takatsu, T., Suzuki, K. et al. (2019). Annual and seasonal changes in the assemblage of planktonic copepods and appendicularians in Funka Bay before and after intrusion of Coastal Oyashio. *Water Fish. Sci.*, 85, 1077–1087.

Shift from smaller to larger species of copepods and appendicularians after intrusion of colder water into the Funka Bay, Japan, 2014-2017.

132. Yvon-Durocher, G., Montoya, J.M., Trimmer, M. & Woodward, G. (2011). Warming alters the size spectrum and shifts the distribution of biomass in freshwater ecosystems. *Glob. Change Biol.*, 17, 1681–1694.

No effect on zooplankton size and community structure in warmed mesocosms.

133. Yvon-Durocher, G., Allen, A.P., Cellamare, M., et al. (2015). Five years of experimental warming increases the biodiversity and productivity of phytoplankton. *PLoS Biol.*, 13, e1002324.

No effect of warming in mesocosm experiment on zooplankton body size.

134. Zamora-Terol, S., Kjellerup, S., Swalethorp, R. et al. Population dynamics and production of the small copepod *Oithona* spp. in a subarctic fjord of West Greenland. *Polar*

Biol., 37, 953–965.
Relatively more smaller species in summer, and more nauplii, across the season in a fjord of West Greenland, 2010.

135. Zhang, H., Urrutia-Cordero, P., He, L. et al. (2018). Life-history traits buffer against heat wave effects on predator–prey dynamics in zooplankton. *Glob. Change Biol.*, 24, 4747–4757.
Higher recruitment of copepods in warmer conditions, leading to more of later-developmental stages (nauplii not counted). Decline of rotifers, increase of cyclopoids.

136. Zingel, P., Cremona, F., Nõges, T. et al. (2018). Effects of warming and nutrients on the microbial food web in shallow lake mesocosms, *Eur. J. Protist.*, 64, 1–12.
No clear effect of warming on protozooplankton (heterotrophic nanoflagellates and ciliates) to mesozooplankton biomass in heated mesocosms compared to ambient ones.
